# Parasitic mites alter chicken behaviour and negatively impact animal welfare

**DOI:** 10.1038/s41598-020-65021-0

**Published:** 2020-05-19

**Authors:** Amy C. Murillo, Alireza Abdoli, Richard A. Blatchford, Eamonn J. Keogh, Alec C. Gerry

**Affiliations:** 1 Department of Entomology, University of California, Riverside, CA USA; 2 Department of Computer Science & Engineering, University of California, Riverside, CA USA; 3 Department of Animal Science, Center for Animal Welfare, University of California, Davis, CA USA

**Keywords:** Animal behaviour, Entomology

## Abstract

The northern fowl mite, *Ornithonyssus sylviarum*, is one of the most common and damaging ectoparasites of poultry. As an obligate blood feeding mite, the northern fowl mite can cause anaemia, slower growth, and decreased egg production of parasitized birds. However, the impact of mites or other ectoparasites on hen behaviour or welfare is not well studied. Here, we use activity sensors (three-axis accelerometers) affixed to individual birds to continuously record hen movement before, during, and after infestation with mites. Movements recorded by sensors were identified to specific bird behaviours through a previously trained algorithm, with frequency of these behaviours recorded for individual birds. Hen welfare was also determined before, during, and after mite infestation of hens using animal-based welfare metrics. Northern fowl mites significantly increased hen preening behaviour and resulted in increased skin lesions of infested birds.

## Introduction

Commercial poultry production in the United States is rapidly changing largely due to public concerns about animal welfare. Until recently, ca. 95% of egg layers in this country were housed in wire cages suspended off the ground^1^, but the industry is rapidly shifting toward cage-free housing for egg production^2^, and it is predicted that over the next decade at least 40% of birds will be housed in cage-free alternative housing systems^3^. One aspect driving consumer demand of cage-free eggs is the perceived increase in the health and welfare of poultry housed in cage-free systems. However, animal welfare encompasses many aspects of animal health and behaviour, which can be complex and difficult to optimize^1,4^. For example, while cage-free housing might provide birds greater freedom of movement throughout a poultry house, birds may also have increased exposure to ectoparasites through contact with the ground, other individuals, or with wild birds.

The most common and damaging ectoparasite (external parasite) of commercial poultry in the United States is the northern fowl mite (NFM; *Ornithonyssus sylviarum* [Canestrini & Fanzango]; reviewed in^5^). NFM are permanent ectoparasites that complete their entire life cycle on-host and can infest chickens regardless of housing type^6,7^. NFM require blood meals to complete their life cycle and to reproduce. Birds heavily infested with NFM can suffer up to 6% blood loss per day^8^. Through their feeding, mites induce skin inflammation, irritation, anaemia, and even death as a result of exsanguination^9–13^. Mite-infested birds exhibit decreased egg production and reduced feed conversion efficiency^12,14^. Infestation with mites or other ectoparasites can also contribute to animal discomfort and pain/injury, which may also hinder birds from fulfilling “normal” behaviours. Despite these obvious negative effects to bird health caused by NFM, they are largely ignored in current animal welfare assessments. In the widely adopted Welfare Quality^15^ assessment protocol, only presence or absence of ectoparasites is noted; there is no qualitative or quantitative evaluation of ectoparasite infestation level or impacts to birds. Even the species of ectoparasite infesting an animal is not recorded, though impacts to animal health and production would be expected to vary by parasite species^12,13,16^. In addition to differential impact by parasite species, the degree of impact will also vary based on severity of infestation, previous exposure to ectoparasites, and other factors. Linking degree of ectoparasite infestation to welfare metrics is a knowledge gap to optimizing high standards of animal welfare in poultry production.

Few studies have examined the effect of NFM on the behaviour of chickens, and these studies were limited to caged birds^17,18^. The time investment for traditional behavioural studies (using video-recording or by direct observation) severely limits the number of animals that can be examined as well as the observation period for each animal. In addition, it can be difficult to elucidate chicken behaviours associated with illness or disease as sick birds are more prone to hiding and thus are not as readily observed^19^.

The use of new technologies, such as on-animal activity sensors, can increase behavioural observations in time and space without the risk of human bias or human interference with animal behaviours (reviewed in^20^). On-animal sensors can also increase the number of individual animals tracked, while increasing the tracking period and the sensitivity for detection of behaviours. The increased data that can be obtained using on-animal sensors provides greater statistical power than can typically be achieved in direct observation studies. On-animal sensors are versatile and can be used to measure duration and type of animal movement, individual animal location, and/or body temperature.

Ectoparasite effects on the health and welfare of poultry in cage-free systems have not been evaluated but are important to understand as poultry husbandry practices continue to shift toward cage-free housing, which is expected to increase the diversity of ectoparasites associated with these birds^21^. In the present study, we use on-animal sensors coupled with visual assessment of animal health to evaluate chicken behaviours and welfare metrics in the absence of mites, at low and high mite scores, following mite control, and after a secondary mite infestation.

## Methods

### Chickens

Forty-eight beak trimmed Hy-Line Brown laying hens were obtained from a local poultry facility and subsequently housed at the Poultry Research Facility at the University of California Riverside (UCR) Agricultural Operations. Hens were 22 weeks old at week 1 of the study. Egg-lay began at ca. 19 weeks old, but production parameters were not recorded as part of the study. This study was approved by and conducted in accordance with the University of California Riverside Institutional Animal Care and Use Committee. Two structures (3.8 by 5.8 m) were divided into two separate sections, creating four ‘poultry houses’ each with 12 birds (a ‘flock’). Each poultry house was equipped with water dispensers, feed troughs, and nest boxes, and had bird density which met or exceeded US standards for cage-free production^22^. Straw bedding (5–10 cm in depth) was added to each house at the beginning of the study and was not removed until the study concluded. Lights were kept on a 16:8 (L:D) h cycle. Each hen within a flock was uniquely marked with coloured leg bands for individual bird identification. One bird in flock 3 was removed during week 11 due to injury and was not replaced.

### Mites

Experimental birds were inoculated with northern fowl mites as described by Martin and Mullens^6^. Briefly, northern fowl mites were aspirated using glass pipettes from source birds maintained at the UCR Poultry Research Facility. Approximately 30 adult mites were placed at the base of feathers in the vent region (underside) of each chicken to ensure a similar level of mite exposure to each bird. Mite numbers were expected to rapidly increase from this initial low level as the mite life cycle is short, requiring as few as 5 days to go from egg to adult^23^. Mites were placed onto birds at week 4 (all flocks) and again at weeks 14 and 15 (flocks 3 and 4 only) to reinfest birds treated with acaricide during weeks 9 and 11 as indicated in the study design below. The vent-area feathers of each bird were visually examined and mite density was scored by a single researcher for consistency of counts using the following scoring system (mite score = # mites): 1 = 1–10, 2 = 11–50, 3 = 51–100, 4 = 101–500, 5 = 501–1000, 6 = 1001–10,000, and 7 > 10,000^24^. Mite scores for all birds were tracked weekly from week 4 through week 20.

### Behaviour

**Figure 1:**
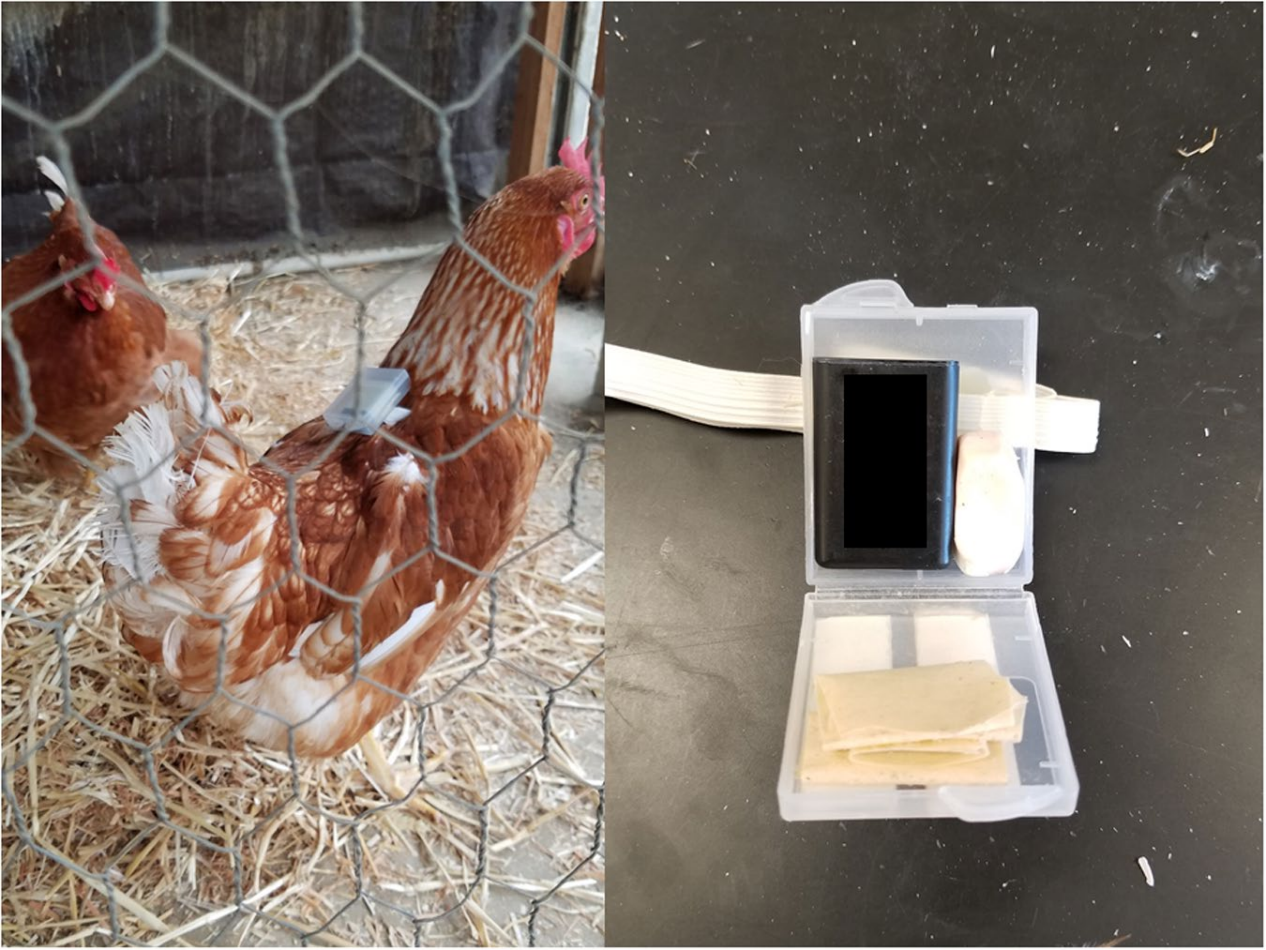
Chicken wearing activity sensor (3-axis accelerometr) in plastic “backpack” (left). Axivity sensor in plastic case (right).

Three-axis accelerometers (“sensors”) (AX3, Axivity Ltd, UK) were used to record the direction and magnitude of acceleration as birds altered body position or moved within the poultry house. Sensors were placed in plastic “backpacks” (Hero 4 AHDBT-401 plastic case, Amazon.com, Seattle, WA, USA) affixed to the back of each bird using elastic bands stretched around the base of each wing (Fig. 1). Sensor data were collected at a rate of 100 Hz (ca. 100 readings/sec).

A “behaviour dictionary” was developed which allowed for the classification of behaviours performed by birds from sensor data^25^. Sensor data used to build and test the behaviour dictionary was collected from 10 different birds (recorded for ≥ 4 hours at a time) over the span of several months. Video recordings of the test birds were synced with sensor output data, and visually observable and distinct behaviours were annotated by a single observer using ELAN open-access software (Max Planck Institute for Psycholinguistics, The Language Archive, Nijmegen, The Netherlands, v. 5.2, https://tla.mpi.nl/tools/tla-tools/elan/).

Three chicken behaviours of interest were identified as distinct, easily observable behaviours associated with chicken welfare and, if they change with ectoparasite infestation, may provide an early indicator of infestation and negative impact to poultry welfare. These behaviours are defined as 1) pecking: bringing the beak to the ground, striking at the ground; 2) preening: manipulating, rearranging, pulling, or smoothing body feathers by the beak; 3) dustbathing: bird is in a sitting or lying position with feathers raised in a vertical wing-shake, including feather-ruffling and shaking^26^. Dustbathing and preening are both important for feather maintenance and thermoregulation^27^. Pecking was used as a proxy for foraging, a behaviour associated with good welfare^28^.

Algorithms to classify bird behaviours were developed from annotated hen activity data^29^. Algorithms were a Nearest-Neighbour Classifier containing one or more examples of each behaviour of interest and utilized a rejection threshold. The reason why we need to allow for multiple examples of each behaviour is that behaviours can be polymorphic. For example, pecking at the ground may manifest differently than pecking at a raised bowl, or a peck may become less frenetic as the bird becomes satiated.

We estimated the accuracy of our algorithm using cross validation, a standard procedure in the machine learning literature, by careful annotation of video that was recorded in parallel with sensor data to test how our model classified each video snippet. The model’s accuracy in distinguishing between the three behaviours of interest, pecking vs. preening vs. dustbathing is near perfect, as these look radically different in the feature space. The more important metric is our recognition accuracy; how accurately we detect a behaviour vs. non-examples of that behaviour, for example pecking vs. non-pecking. Under this metric, our model’s F-score, where $F=2\times \frac{precision\times recall}{precision+recall}$, for the three behaviours of interest is: Dustbathing: 1.00; Preening: 1.00; Pecking: 0.88. Both dustbathing and preening are very distinctive in our feature space and offer few challenges. The pecking behaviour is more difficult, as our algorithm sometimes confuses fast walking movements for pecking. The numbers above reflect a slightly improved version of the algorithm presented in^29^, the improved version of the algorithm is under review and may be accessed here^30^.

### Welfare

Chickens were visually assessed and scored for physical condition of welfare quality based on an adaptation of the Welfare Quality protocol^15^ by Blatchford *et al*.^31^. The Welfare Quality assessment is a frequently used research tool with a validated set of measures used as a basis for recording animal welfare conditions^32^. Briefly, each bird was examined for abnormal condition of the eyes, comb, beak, keel, feet, toes, skin and feather condition (Table 1). All metrics were scored as a 0, 1, or 2 (0 = absent, 1 = present and moderate, 2 = present and severe). Flocks were pooled for welfare analyses at weeks 1, 4, 7, and 12. At week 19, flocks 1 and 2 were mite-free while flocks 3 and 4 were mite infested; at this time point flocks were pooled by mite status for analysis.

**Table 1:** Welfare Quality protocol metrics were scored for each bird at weeks 1, 4, 7, 12, and 19.

	Description
Eyes	0: Absence of abnormalities
	1: Presence of abnormalities
Nose	0: Absence of abnormalities
	1: Presence of abnormalities
Comb	0: No wounds present
	1: <3 fresh pecks or scratches present
	2: ≥3 fresh pecks or scratches present
Beak	1: Beak trimmed with mild abnormalities
	2: Beak trimmed with severe abnormalities
Feather Loss	0: None present
	1: Present; <5 cm in diameter
	2: Present; >5 cm in diameter
Foot Abnormalities	0: None present
	1: Small lesions or skin thickening present
	2: Foot swelling visible from dorsal side
Toe Damage	0: None present
	1: Broken toe or missing claw present
Keel Bone Damage	0: None present
	1: Lumps or deviations present
Soiled Feathers	0: No manure in vent feathers
	1: Moderate soiling
	2: Severe soiling
Skin Lesions	0: None present
	1: Present; ≤2 cm in diameter
	2: Present; >2 cm

Metrics were scored as normal (score = 0), present and moderate (score = 1) or present and severe (score = 2) if applicable.

### Study design

**Figure 2:**
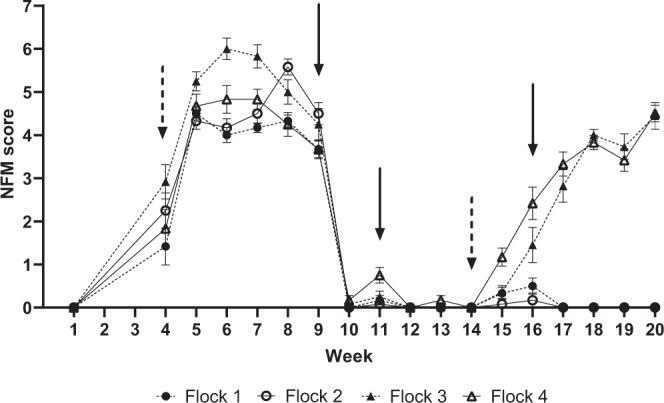
Average (±SE) mite scores for each chicken flock. All birds were uninfested at week 1, but 45 of 48 birds were unintentionally (naturally) infested with a low number of mites at an undetermined time prior to week 4 when birds were next examined for mites and the study was initiated by deliberate infestation of birds with mites. Black dashed arrows indicate when mites were added to individual birds in all flocks at week 4, and to flocks 3 & 4 only at week 14. Black solid arrows indicate weeks when acaricide treatments were applied to all flocks (weeks 9 and 11) or to flocks 1 & 2 only (week 16). The mite scoring system used (score = # NFM per bird) is as follows: 1 = 1–10, 2 = 11–50, 3 = 51–100, 4 = 101–500, 5 = 501–1000, 6 = 1001–10,000, and 7 > 10,000.

The study was conducted over 20 wk from November 2017 to April 2018. On a single date during week 1, week 4, and then weekly thereafter each chicken in all four poultry flocks was visually examined and scored for mites (Table 2). Birds were initially free of mites in week 1 but were unintentionally infested with a low level of NFM by week 4 (45/48 birds infested) (Fig. 2). After week 4 mite scoring, all birds were deliberately infested with additional NFM (as described above) to ensure that all birds in each flock were exposed to a similar infestation level of mites from weeks 5–9. At weeks 9 and 11 all birds were treated with an acaricide, RaVap (Bayer, Shawnee Mission, Kansas, USA), following label instructions to eliminate mites. At week 14, birds in flocks 3 and 4 only were reinfested with NFM, leaving flocks 1 and 2 as comparative controls for the remainder of the study. Flocks 1 and 2 were treated with RaVap again at week 16 to ensure they remained uninfested through the end of the study at week 20.

**Table 2:** Study schedule.

	Study Week																			
**Study Activity**	1	2	3	4	5	6	7	8	9	10	11	12	13	14	15	16	17	18	19	20
NFM scored	X			X	X	X	X	X	X	X	X	X	X	X	X	X	X	X	X	X
NFM added to birds				X^*^										X^†^	X^†^					
Welfare Assessment	X			X			X					X							X	
Behaviours recorded	X			X			X					X							X	
Acaricide Treatment									X		X					X^ǂ^				

N = 48 or 47 total birds (wk 1–11 or wk 12–20, respectively) separated into 4 flocks; ^*^all birds infested w/ NFM at week 4; ^†^NFM added to flock 3 & 4 only; ^ǂ^treatment applied to flock 1 & 2 only.

After recording mite scores during weeks 1, 4, 7, 12, and 19, each chicken was also scored for welfare quality metrics, then fitted with a sensor to record bird behaviour for 1 wk (Table 2). After each 1-wk recording period, data were downloaded from each sensor and run through the developed algorithm described above^25,28^ to identify and tally each behaviour event recorded for the defined behaviours of interest. Detailed sensor data are provided as supplementary material (Supplementary 1) and are also available here^25^.

### Statistical analyses

Statistical analyses were performed using SAS software (SAS Institute Inc., Cary, NC, 2012, v. 9.4), with PROC MEANS used to generate means and standard errors for mite score and behaviour events.

To determine whether bird behaviours differed across time for each flock, a general linear model (PROC GLM) was used with number of behaviour events as the response variable and study week as the independent variable, with means separated by Tukey HSD for each behaviour.

Differences in the number of behaviour events over time were further analysed using mixed-model repeated-measures (PROC MIXED) with NFM score, flock, and bird ID (subject) as fixed effects and study week as a random effect. Initially a comprehensive model was used, but subsequent analyses were separated by flock when significant differences by flock were found.

Welfare metric scores were ordinal, therefore nonparametric tests (PROC NPAR1WAY) were used for analyses. All flocks were pooled for welfare analyses, except for week 19 data when flock 1 & 2 (uninfested flocks) were pooled separately from flock 3 & 4 (NFM-infested flocks). Each study week was separated for analysis. Kruskall-Wallis tests were used to determine whether welfare metric scores varied by NFM infestation for each welfare metric (comb wounds, skin lesions, or soiled feathers) with prevalence or severity as the response variable and NFM score as the class variable. Mann-Whitney tests were used to make comparisons for each welfare metric between week 1 (no mites) as compared to weeks 4 (low mites), 7 (high mites), 12 (post-trt, no mites), or 19 (post-trt, F3&4 w/mites). Welfare metric prevalence was the response variable and week was the class variable.

## Results

### Mite abundance

In general, mite scores increased rapidly following intentional infestation of chickens with mites in week 4, reaching a peak in weeks 5–6, and then remaining consistently high until acaricide treatment at week 9 (Fig. 2). Following acaricide treatment, mite scores remained low in subsequent control flocks (flocks 1 & 2) through the remainder of the study. In reinfested flocks (flocks 3 & 4), mite scores rose steadily after mite reintroduction to birds at week 14, reaching a peak mite score in weeks 18–20 of similar magnitude to the early infestation. Average mite scores at welfare assessment weeks were: week 1 = 0.0 ± 0.0; week 4 = 2.10 ± 0.22; week 7 = 4.83 ± 0.14; week 12 = 0.0 ± 0.0; week 19 = 0.0 ± 0.0 (flocks 1 & 2) or 3.57 ± 0.20 (flocks 3 & 4).

### Pecking

**Figure 3:**
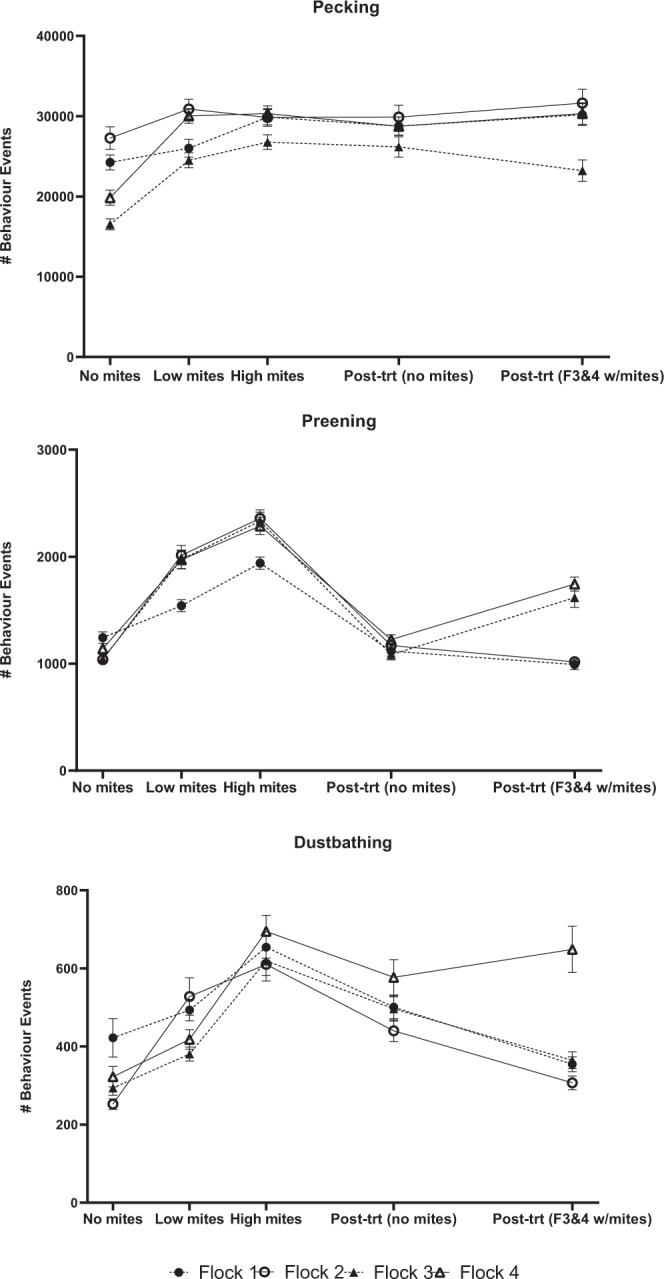
Recorded pecking, preening, and dustbathing behaviours (means ± SE) for each of four flocks as recorded by on-animal sensors. The mite status at each week is as follows: week 1 = no mites, week 4 = low mites, week 7 = high mites, week 12 = treated; no mites, week 19 = no mites in flocks 1 & 2, mites present in flocks 3 & 4.

**Figure 4:**
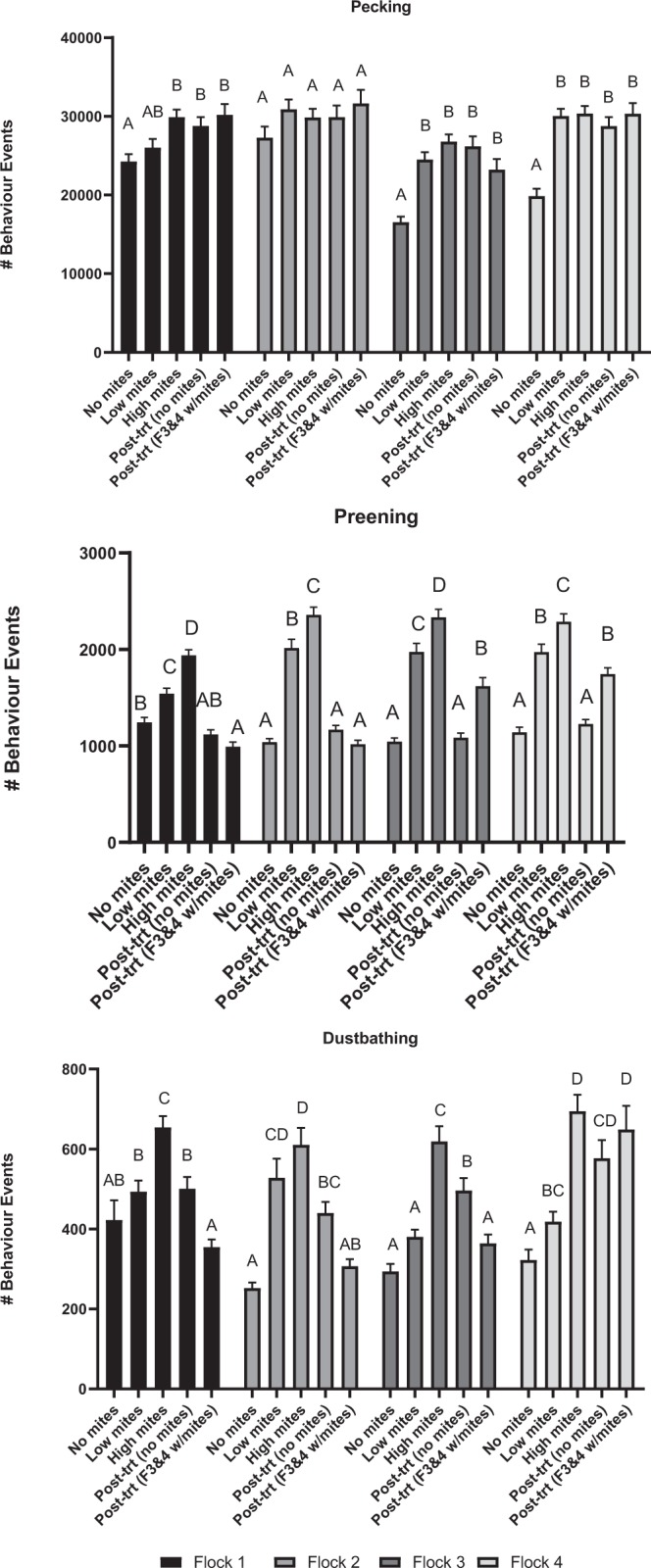
Behaviour events (mean ± SE) by week for each flock. Bars with the same letter are not significantly different within a flock (P > 0.05). The mite status at each week is as follows: week 1 = no mites, week 4 = low mites, week 7 = high mites, week 12 = treated; no mites, week 19 = no mites in flocks 1 & 2, mites present in flocks 3 & 4.

Pecking behaviours were similar for all four flocks over time. Pecking behaviour generally increased from week 1 to week 4 for three of four flocks (Flock 1: F = 5.16, df = 4, P = 0.0005; Flock 2: F = 1.28, df = 4, P = 0.278; Flock 3: F = 14.47, df = 4, P < 0.0001; Flock 4: F = 16.07, df = 4, P < 0.0001) with no further differences among subsequent weeks for any flock regardless of mite infestation level (Figs. 3 and 4)

### Preening

Preening significantly increased from week 1 through week 7 (high mites) for each flock (Flock 1: F = 52.47, df = 4, P < 0.0001; Flock 2: F = 89.57, df = 4, P < 0.0001; Flock 3: F = 56.54, df = 4, P < 0.0001; Flock 4: F = 50.74, df = 4, P < 0.0001) (Figs. 3 and 4). At week 19, mite-infested flocks 3 & 4 exhibited more preening than the uninfested control flocks 1 & 2 (F = 9.73, df = 3, P < 0.0001). The number of preening events was positively associated with mite scores in all four flocks (Flock 1: F = 11.53, df = 5, P < 0.0001; Flock 2: F = 10.49, df = 5, P < 0.0001; Flock 3: F = 4.68, df = 7, P = 0.0005; Flock 4: F = 5.17, df = 6, P = 0.0003).

### Dustbathing

Dustbathing behaviours increased from week 1 through week 7 (high mites), peaked and then declined over the remainder of the study, except for flock 4 which had an increase in dustbathing after mites were reintroduced to flocks 3 & 4 at week 14 (Figs. 3 and 4) (Flock 1: F = 12.92, df = 4, P < 0.0001; Flock 2: F = 18.15, df = 4, P < 0.0001; Flock 3: F = 23.01, df = 4, P < 0.0001; Flock 4: F = 13.40, df = 4, P < 0.0001).

### Welfare

Throughout the study all welfare metrics observed other than those related to comb, skin, and feather condition were consistently scored as normal (score = 0) or were rarely scored as abnormal (score = 1 or 2) and were thus not further analysed. In contrast, the welfare conditions of comb wounds, skin lesions, and feather soiling were more often scored as abnormal and are therefore analysed below relative to levels of mite infestation. The complete welfare metrics data set is provided as supplementary material (Supplementary 2).

**Figure 5:**
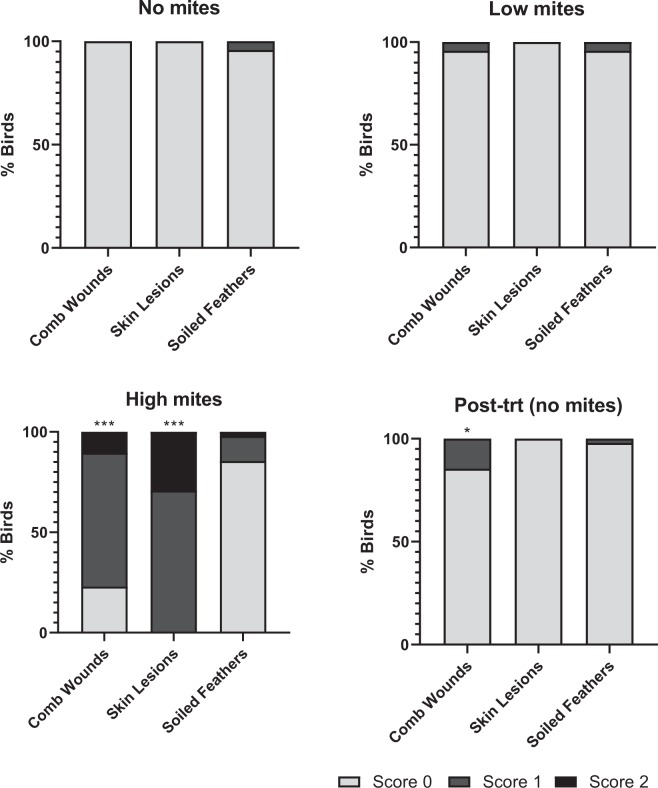
Welfare scores for all flocks when there were no mites (week 1), low mites (week 4), high mites (week 7) and after treatment (week 12). Coloured bars indicate welfare scores for each metric as follows: light grey = 0 (normal); grey = 1 (present and moderate); black = 2 (present and severe). Significant differences for presence/absence welfare metrics during weeks 4, 7, and 12 compared to week 1 are indicated by (*) P ≤ 0.01; (**) P ≤ 0.001; (***) P ≤ 0.0001.

Welfare metrics recorded during week 1 of the study, when all birds were free of mites, were used as a baseline for comparison of welfare throughout the rest of the study as mite numbers fluctuated. During week 1, birds were free of comb wounds and skin lesions, and only 2/48 birds showed any feather soiling (Fig. 5).

### Comb wounds

**Figure 6:**
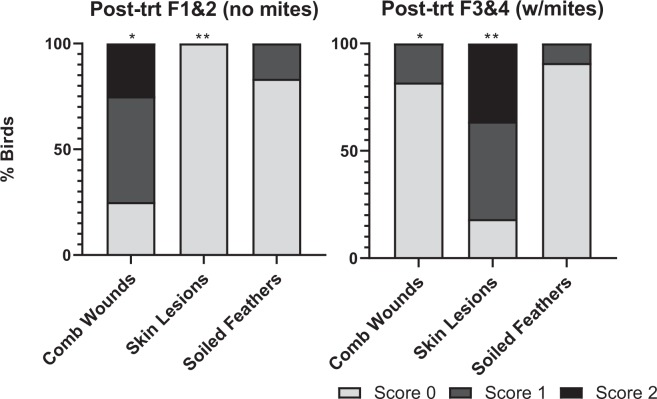
Flock-level welfare metric scores at week 19. Birds in flocks 1 & 2 (left) were uninfested, while birds in flocks 3 & 4 (right) were mite-infested. Bars indicate welfare scores for each metric as follows: light grey = 0 (normal); grey = 1 (moderate abnormality); black = 2 (severe abnormality). Significant differences for presence/absence welfare metrics between flocks 1 & 2 (no mites) and flocks 3 & 4 (mites) are indicated by (*) P ≤ 0.01; (**) P ≤ 0.001.

Relative to the week 1 (no mites) baseline data, there was a not significant increase in prevalence of comb wounds with 2/48 birds having comb wounds when mite numbers were low at week 4 (X^2^ = 2.02, df = 1, P = 0.1551). This was followed by a significant increase in comb wounds to 37/48 birds when mite numbers were high at week 7 (X^2^ = 2.02, df = 1, P = 0.1551), including 32 birds with moderate wounds and 5 birds with severe wounds. However, mite score was not related to severity of comb wounds at week 7 (X^2^ = 5.25, df = 3, P = 0.154). Only 7/47 birds had comb wounds (all moderate) in week 12 after mites had been eliminated from flocks, though this was still a higher comb wound prevalence relative to week 1 (X^2^ = 7.47, df = 1, P = 0.0063). At week 19, after flocks 3 & 4 only had been reinfested, both mite-free and mite-infested flocks showed higher comb wound prevalence relative to week 1 (X^2^ = 21.36, df = 1, P < 0.0001 and X^2^ = 5.71, df = 1, P = 0.0168, respectively), but prevalence and severity was not as expected if mite infestation was related to comb wounds as, surprisingly, mite-free flocks showed higher prevalence of comb wounds (15/24 birds; 12 moderate and 3 severe) than mite-infested flocks (5/23 birds; 3 moderate and 2 severe) (X^2^ = 7.8, df = 1, P = 0.005) (Fig. 6).

### Skin lesions

**Figure 7:**
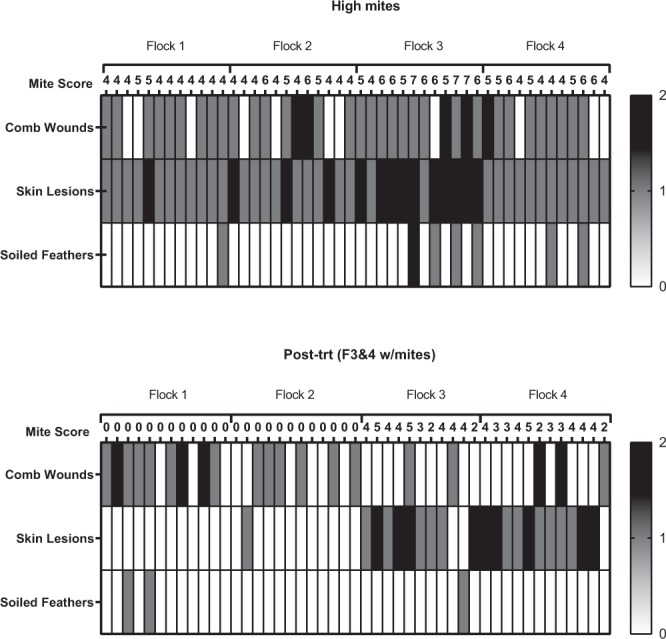
Heat map of welfare metrics (0, 1, or 2) for each bird when mites were high (week 7) and when flocks 1 & 2 were uninfested and flocks 3 & 4 were mite-infested (week 19). .

All birds were free of skin lesions at week 1 (no mites) and week 4 (low mites). At week 7 all birds exhibit skin lesions (14 moderate and 34 severe), an increase relative to week 1 (X^2^ = 95.00, df = 1, P < 0.0001). Mite score was related to skin lesion severity (X^2^ = 14.01, df = 3, P = 0.0029) with higher mite scores associated with greater severity of skin lesions (Fig. 7). At week 12, just three weeks following the first mite treatment, all birds had healed, and no skin lesions were observed. At week 19, five weeks after mite-reinfestation to flocks 3 & 4, 21/23 mite-infested birds exhibit skin lesions (12 moderate and 9 severe). In contrast, only one mite-free bird in flocks 1 & 2 had any skin lesions, a significant difference in prevalence between mite-free and mite-infested flocks (X^2^ = 35.96, df = 3, P < 0.0001) (Fig. 6). There were no differences in skin lesion severity among mite-infested birds (i.e. mite infestation level did not impact skin lesion severity) (X^2^ = 5.18, df = 3, P = 0.159).

### Soiled feathers

The number of birds with soiled feathers between week 1 (baseline) and week 4 (low mites) was the same, with only 2/48 birds with soiled feathers (Fig. 5). This increased to 7/48 birds when mite levels were high (week 7), with 6 moderate and 1 severe case. This was not statistically different from week 1 soiled feather prevalence (X^2^ = 3.03, df = 1, P = 0.0816). However, mite score did relate to the severity of feather soiling when mites were high (X^2^ = 12.8, df = 3, P = 0.005) (Fig. 7). After mite treatment at week 12, only one bird had soiled feathers. This barely increased at week 19, where 2/24 mite-free and 1/23 mite-infested birds exhibited soiled feathers (all moderate).

## Discussion

On-animal sensors have been used to track or describe the behaviours performed by a variety of animals including livestock (e.g.^26,33–36^. In cattle, this technology has been used to track herd movements^33^ including movement of animals exposed to biting flies^34^. For poultry, sensors have been developed to help with surveillance for high pathogenic avian influenza by detecting periods of below-average activity for birds, a sign of illness^37^. Behaviour has also been used as a monitoring tool for food-borne pathogens of broilers (meat birds)^38^. The Axivity accelerometers used in the current study were developed for use on human subjects and were advantageous because of their small size, light weight, and waterproof case. The accelerometers also record the data locally (on the device) which eliminates the need for subjects to remain close to a receiver (that collects the data remotely) and reduces the risk of lost data (that can occur when using a receiver). At 100 Hz the Axivity accelerometers can record up to 14 days of data uninterrupted which allowed us to record very large data sets for each individual bird. Published resources exist for analysing or describing animal behaviours from collected accelerometer data e.g.^39–41^. The present study uses a unique approach of analysing shape and feature of accelerometer data to create the behaviour dictionary which describes specific behaviours rather than describing general animal movements^28^. All of these materials, including software with a user-friendly interface, are now available for researchers to use^32^.

Flocks exhibited similar welfare metrics at week 1 (no mites) and week 4 (low mites), suggesting that a low initial mite score does not substantially impact bird welfare, or that impacts are too low to observe using the current welfare metric scoring system. By week 7, welfare impacts were noted with high prevalence and severity of skin lesions being positively associated with increasing mite score. Skin lesions disappeared (healed) by week 12 after elimination of mites with an acaricidal treatment. Following reinfestation of flocks 3 & 4 with mites at week 14, birds in these flocks again showed increased prevalence of skin lesions relative to mite-free flocks near the end of the study (week 19), providing further support that high mite numbers are associated with increased prevalence and severity of skin lesions on infested birds. Skin lesions are likely related to the bird’s immune response to mite feeding in the vent region^13^ where all skin lesions were observed. All birds were beak trimmed, a common industry practice^42^, so skin lesions detected were not likely caused by preening behaviour, though this behaviour also increased for birds as mite infestation levels increased.

Preening behaviour was positively influenced by NFM scores, with the greatest number of preening events recorded when mite populations were highest in all flocks at week 7. Vezzoli *et al*.^18^ investigated the effects of NFM infestation on chicken preening and found that NFM-infested birds did not spend more time preening overall, but rather that preening became more concentrated in the vent area. In the present study, we cannot differentiate where preening behaviours were directed, just that the behaviour increased over time. Increased preening in the vent area may be a direct response to mite biting, or an indirect response to mite feeding which can cause skin inflammation^13^ and may also initiate a histamine response resulting in an itch sensation^43^. While Vezzoli *et al*.^18^ did not find an increase in preening behaviour in mite-infested chickens, these authors recorded preening behaviour only over a couple of hours during a single time of day (afternoon) and additionally birds were held in cages, which may have restricted their behaviours. In contrast, the present study showed an increase in preening behaviour for mite-infested chickens probably due to the much greater volume of data for analysis that was available by using sensors applied to individual birds and by capturing behaviour data constantly over several days’ time.

Dustbathing is a complex innate behaviour typically performed by each bird for 30 min every other day^44^ to keep feathers in good condition^26,45^. Chickens exhibit dustbathing behaviour at a young age when given access to appropriate substrate, and expression of this behaviour is maintained throughout the life of the bird. However, external factors can have a strong effect on expression of this behaviour causing changes in frequency and duration. Dustbathing may be socially facilitated^46^ and variation among individuals may be influenced by social hierarchy^47^. In addition, dustbathing behaviour by individual birds is known to vary from week to week^7^. Dustbathing is a circadian behaviour that occurs infrequently, and data collection that is not restricted to a single time of day is best for sampling this type of behaviour^48^. In the present study dustbathing behaviour was greatest at week 7 when mite scores were highest. Following the elimination of mites from birds, dustbathing was reduced in all flocks until mites were reintroduced to flocks 3 & 4 at week 14, whereupon dustbathing increased again in flock 4 but not in flock 3. The cause of this difference in dustbathing between the reinfested flocks is unknown and may be due to some environmental condition that was not detectable or observed. A previous study found no effect of NFM infestation on frequency or duration of dustbathing behaviour^17^, however this study was limited to caged birds. While, increasing NFM was associated with greater dustbathing during the first part of the current study, the lack of consistency in this relationship following mite reinfestation of flocks 3&4 indicates that mite impacts on dustbathing behaviour needs further examination.

Pecking was a stable behaviour that did not change much over time after week 4. The small increase in pecking behaviour from week 1 to week 4 is perhaps explained by increased need for food consumption as birds increased in body size with age particularly during the earliest weeks of the study. Increases in preening and dustbathing through week 7 were not associated with a decrease in pecking behaviours. Future work can help determine if mite-driven behaviours are influencing normal periods of rest or inactivity for birds. This may be an important step to understanding overall mite effects on chicken well-being, energy expenditure, and economic output. This may be especially important for organic or pasture-raised birds that have the ability to spend time outside and provide important ecosystem services, such as pasture fertilization and pest management, particularly when chickens are used as part of a multi-animal species pasture rotation system^4^.

Parasites may impose behavioural and energy costs on their hosts. For example, wild birds with high nematode parasite loads had reduced flight time relative to birds with lower parasite loads, perhaps as a result of the higher energy expenditure associated with flight activity for heavily parasitized birds^49^. If maximum energy expenditure remains constant even under stress of parasitaemia, as suggested by^49^, then any increase in specific chicken behaviours as a result of increasing mite density would be offset by decreases in other activities or decreased duration or intensity of behaviours. In the current study, we did not determine energy expenditures of mite-parasitized or unparasitized chickens, nor did we monitor the duration or intensity of behaviours other than those discussed in this study which were anticipated to perhaps be impacted by parasitaemia. Also, since chickens in the current study were provided food ad libitum, perhaps any additional energy expenditure associated with parasitaemia could be compensated for by increased feed consumption. A previous study found no effect of NFM infestation on laying hen resting metabolic rate, though feed intake relative to egg output was significantly affected by the presence of mites (i.e. feed intake increased)^50^.

In this study, individual animal behaviours were highly variable from day-to-day and by individual, but patterns did emerge among flocks, particularly related to mite infestation level. NFM infestation was shown to increase hen preening behaviour and to increase the presence of skin lesions which are important as a measure of animal welfare. Use of on-animal sensors allows for the collection of very large datasets and the ability to track behaviours continuously for several days which can mitigate the effects of limited sampling interval and duration typical when using direct observation of animals by researchers in behaviour studies^48^. On-animal sensors allow for continuous data collection without the time and labour commitment of traditional observational studies. Future studies will likely move toward use of sensors that record accelerometer data in real-time to make instantaneous observations of animal behaviour allowing for real-time decisions regarding animal health or management needs.

## Supplementary information

Supplementary information.

Supplementary information2.
