# Supplementary material for: Parasitic mites alter chicken behaviour and negatively impact animal welfare
Source: Sci Rep. 2020 May 19;10:8236. doi: 10.1038/s41598-020-65021-0 (PMC7237419; doi:10.1038/s41598-020-65021-0)
Supplement: Supplementary file 1 — Supplementary information. [file 41598_2020_65021_MOESM1_ESM.pdf]

Supplementary Table 1 to:

Parasitic mites alter chicken behaviour and negatively impact animal welfare

Amy C. Murillo*[1], Alireza Abdoli[2], Richard A. Blatchford[3], Eamonn J. Keogh[2], and Alec C. Gerry[1]

*Corresponding author: Amy Murillo, Dept. of Entomology, Univ. of California, Riverside, CA, 92521, USA +1 (951) 827-5741, amy.murillo@ucr.edu

[1] Department of Entomology, University of California, Riverside, CA

[2] Department of Computer Science & Engineering, University of California, Riverside, CA

[3] Department of Animal Science, Center for Animal Welfare, University of California, Davis, CA

Week	Date	Flock	Bird	Pecking	Preening	Dustbathing
1	11/22/2017	1	1	10353	1185	397
1	11/23/2017	1	1	19358	1312	675
1	11/24/2017	1	1	23868	1069	623
1	11/25/2017	1	1	20281	1628	842
1	11/26/2017	1	1	24923	1542	1357
1	11/27/2017	1	1	22851	1631	1116
1	11/28/2017	1	1	29706	1613	1573
1	11/22/2017	1	2	18954	693	194
1	11/23/2017	1	2	26005	713	103
1	11/24/2017	1	2	23908	687	176
1	11/25/2017	1	2	24718	680	200
1	11/26/2017	1	2	24843	776	226
1	11/27/2017	1	2	28341	661	287
1	11/28/2017	1	2	35767	592	276
1	11/22/2017	1	3	8940	1894	171
1	11/23/2017	1	3	25059	1018	183
1	11/24/2017	1	3	35294	1119	227
1	11/25/2017	1	3	31699	1025	323
1	11/26/2017	1	3	27686	1002	200
1	11/27/2017	1	3	24198	1103	342
1	11/28/2017	1	3	21153	1111	192
1	11/22/2017	1	4	7371	2805	176
1	11/23/2017	1	4	14113	1958	217
1	11/24/2017	1	4	16968	1750	188
1	11/25/2017	1	4	24783	1489	178
1	11/26/2017	1	4	20911	1045	162
1	11/27/2017	1	4	21178	1710	186
1	11/28/2017	1	4	21458	1620	167
1	11/22/2017	1	5	5510	1643	265
1	11/23/2017	1	5	13826	963	105
1	11/24/2017	1	5	14203	929	304
1	11/25/2017	1	5	12204	1003	214
1	11/26/2017	1	5	13830	1343	219
1	11/27/2017	1	5	15577	1172	279
1	11/28/2017	1	5	21660	1573	344
1	11/22/2017	1	6	4214	842	201
1	11/23/2017	1	6	10167	565	154
1	11/24/2017	1	6	12981	671	441
1	11/25/2017	1	6	21402	753	324
1	11/26/2017	1	6	19390	856	324
1	11/27/2017	1	6	23421	1038	600
1	11/28/2017	1	6	23720	1211	545
1	11/22/2017	1	7	22015	1082	267
1	11/23/2017	1	7	30945	1192	375
1	11/24/2017	1	7	26097	1548	547
1	11/25/2017	1	7	23053	1708	511
1	11/26/2017	1	7	29748	1647	479

1	11/27/2017	1	7	29459	2059	593
1	11/28/2017	1	7	33169	1570	647
1	11/22/2017	1	8	8635	2193	179
1	11/23/2017	1	8	18326	1489	127
1	11/24/2017	1	8	37985	1256	144
1	11/25/2017	1	8	27490	1420	171
1	11/26/2017	1	8	26701	1467	390
1	11/27/2017	1	8	29364	1476	338
1	11/28/2017	1	8	28702	1387	581
1	11/22/2017	1	9	16914	2147	367
1	11/23/2017	1	9	26466	1268	568
1	11/24/2017	1	9	26286	1644	1883
1	11/25/2017	1	9	25835	2139	1193
1	11/26/2017	1	9	28843	2359	1936
1	11/27/2017	1	9	27583	2018	1861
1	11/28/2017	1	9	28524	2005	2407
1	11/22/2017	1	10	21628	1905	175
1	11/23/2017	1	10	39409	973	205
1	11/24/2017	1	10	43139	971	234
1	11/25/2017	1	10	33865	1010	137
1	11/26/2017	1	10	36574	1051	283
1	11/27/2017	1	10	31176	1149	267
1	11/28/2017	1	10	36746	1211	282
1	11/22/2017	1	11	25445	953	144
1	11/23/2017	1	11	40665	780	139
1	11/24/2017	1	11	31298	747	106
1	11/25/2017	1	11	24571	773	163
1	11/26/2017	1	11	38116	790	147
1	11/27/2017	1	11	35728	1432	234
1	11/28/2017	1	11	42660	948	246
1	11/22/2017	1	12	10130	489	147
1	11/23/2017	1	12	26365	442	216
1	11/24/2017	1	12	32367	714	311
1	11/25/2017	1	12	22642	661	463
1	11/26/2017	1	12	23833	636	316
1	11/27/2017	1	12	18353	964	281
1	11/28/2017	1	12	23134	774	154
1	11/22/2017	2	13	21732	1197	157
1	11/23/2017	2	13	31856	823	103
1	11/24/2017	2	13	36614	917	144
1	11/25/2017	2	13	42157	975	174
1	11/26/2017	2	13	39433	714	208
1	11/27/2017	2	13	42908	1082	198
1	11/28/2017	2	13	44001	1261	244
1	11/22/2017	2	14	6310	946	176
1	11/23/2017	2	14	9746	840	197
1	11/24/2017	2	14	12928	768	186
1	11/25/2017	2	14	15364	693	433

1	11/26/2017	2	14	13191	824	170
1	11/27/2017	2	14	16355	828	273
1	11/28/2017	2	14	14582	824	306
1	11/22/2017	2	15	20762	685	236
1	11/23/2017	2	15	37444	640	177
1	11/24/2017	2	15	37530	635	235
1	11/25/2017	2	15	40934	821	269
1	11/26/2017	2	15	34427	863	382
1	11/27/2017	2	15	41668	906	402
1	11/28/2017	2	15	39061	926	457
1	11/22/2017	2	16	7378	1298	181
1	11/23/2017	2	16	19719	856	167
1	11/24/2017	2	16	23557	868	289
1	11/25/2017	2	16	34097	915	308
1	11/26/2017	2	16	30724	1148	293
1	11/27/2017	2	16	36112	1182	208
1	11/28/2017	2	16	45784	1152	247
1	11/22/2017	2	17	15065	1493	156
1	11/23/2017	2	17	29750	1074	240
1	11/24/2017	2	17	32867	1170	367
1	11/25/2017	2	17	37232	1227	391
1	11/26/2017	2	17	32559	1306	322
1	11/27/2017	2	17	33519	1520	517
1	11/28/2017	2	17	38525	1123	351
1	11/22/2017	2	18	17966	1573	155
1	11/23/2017	2	18	33972	1267	134
1	11/24/2017	2	18	30429	1947	293
1	11/25/2017	2	18	25349	1760	202
1	11/26/2017	2	18	32881	1174	184
1	11/27/2017	2	18	28901	1641	301
1	11/28/2017	2	18	33133	1239	438
1	11/22/2017	2	19	9955	1298	271
1	11/23/2017	2	19	14520	963	74
1	11/24/2017	2	19	17744	1216	133
1	11/25/2017	2	19	15256	1020	369
1	11/26/2017	2	19	16060	1440	87
1	11/27/2017	2	19	12803	1358	262
1	11/28/2017	2	19	23739	1174	181
1	11/22/2017	2	20	23211	1641	226
1	11/23/2017	2	20	44793	1208	227
1	11/24/2017	2	20	42132	1107	403
1	11/25/2017	2	20	46033	1360	719
1	11/26/2017	2	20	48159	988	234
1	11/27/2017	2	20	50734	1422	362
1	11/28/2017	2	20	55323	1290	620
1	11/22/2017	2	21	7026	972	136
1	11/23/2017	2	21	11787	649	107
1	11/24/2017	2	21	16319	598	141

1	11/25/2017	2	21	20102	492	210
1	11/26/2017	2	21	16564	563	136
1	11/27/2017	2	21	17796	678	186
1	11/28/2017	2	21	18156	640	170
1	11/22/2017	2	22	13193	697	217
1	11/23/2017	2	22	20844	795	91
1	11/24/2017	2	22	26634	875	164
1	11/25/2017	2	22	30161	817	142
1	11/26/2017	2	22	30110	1207	201
1	11/27/2017	2	22	28432	1394	175
1	11/28/2017	2	22	25913	1228	255
1	11/22/2017	2	23	17325	2025	231
1	11/23/2017	2	23	34546	911	250
1	11/24/2017	2	23	48204	867	312
1	11/25/2017	2	23	51666	986	535
1	11/26/2017	2	23	45134	912	373
1	11/27/2017	2	23	46960	941	483
1	11/28/2017	2	23	41621	860	443
1	11/22/2017	2	24	4741	2087	157
1	11/23/2017	2	24	13495	1094	98
1	11/24/2017	2	24	15945	421	209
1	11/25/2017	2	24	8026	437	238
1	11/26/2017	2	24	14847	463	109
1	11/27/2017	2	24	14106	407	214
1	11/28/2017	2	24	12610	540	193
1	11/22/2017	3	25	9742	875	163
1	11/23/2017	3	25	16282	498	134
1	11/24/2017	3	25	16007	583	137
1	11/25/2017	3	25	20273	783	231
1	11/26/2017	3	25	20164	695	97
1	11/27/2017	3	25	23257	852	248
1	11/28/2017	3	25	19790	1592	102
1	11/22/2017	3	26	7521	1008	125
1	11/23/2017	3	26	16171	1068	199
1	11/24/2017	3	26	15849	1110	595
1	11/25/2017	3	26	17293	1475	508
1	11/26/2017	3	26	22588	1328	488
1	11/27/2017	3	26	23000	1664	711
1	11/28/2017	3	26	23005	1845	1210
1	11/22/2017	3	27	5275	902	175
1	11/23/2017	3	27	10311	851	402
1	11/24/2017	3	27	15423	702	303
1	11/25/2017	3	27	21158	849	464
1	11/26/2017	3	27	18627	1070	314
1	11/27/2017	3	27	26132	1205	250
1	11/28/2017	3	27	23251	1179	227
1	11/22/2017	3	28	6307	629	100
1	11/23/2017	3	28	14054	701	142

1	11/24/2017	3	28	18642	640	156
1	11/25/2017	3	28	23935	743	181
1	11/26/2017	3	28	17630	794	128
1	11/27/2017	3	28	33107	793	232
1	11/28/2017	3	28	33908	709	154
1	11/22/2017	3	29	4977	432	191
1	11/23/2017	3	29	7865	876	270
1	11/24/2017	3	29	10276	774	273
1	11/25/2017	3	29	8825	996	308
1	11/26/2017	3	29	10817	964	254
1	11/27/2017	3	29	16237	804	471
1	11/28/2017	3	29	13058	1218	293
1	11/22/2017	3	30	8274	1059	97
1	11/23/2017	3	30	21757	869	124
1	11/24/2017	3	30	24993	1125	225
1	11/25/2017	3	30	22764	870	288
1	11/26/2017	3	30	20474	1023	203
1	11/27/2017	3	30	26910	1118	303
1	11/28/2017	3	30	26328	912	235
1	11/22/2017	3	31	6264	523	114
1	11/23/2017	3	31	11338	552	234
1	11/24/2017	3	31	15955	775	302
1	11/25/2017	3	31	17140	729	321
1	11/26/2017	3	31	18032	798	419
1	11/27/2017	3	31	20935	1059	625
1	11/28/2017	3	31	19647	952	420
1	11/22/2017	3	32	10554	783	129
1	11/23/2017	3	32	19528	878	209
1	11/24/2017	3	32	18470	704	210
1	11/25/2017	3	32	13767	756	283
1	11/26/2017	3	32	20728	677	198
1	11/27/2017	3	32	18178	822	352
1	11/28/2017	3	32	21390	902	252
1	11/22/2017	3	33	7574	1344	157
1	11/23/2017	3	33	12835	1483	187
1	11/24/2017	3	33	14678	1129	244
1	11/25/2017	3	33	13911	1072	454
1	11/26/2017	3	33	13938	1139	185
1	11/27/2017	3	33	20142	1597	475
1	11/28/2017	3	33	14805	1376	254
1	11/22/2017	3	34	9711	1156	129
1	11/23/2017	3	34	21938	932	216
1	11/24/2017	3	34	20522	787	383
1	11/25/2017	3	34	25082	943	286
1	11/26/2017	3	34	25317	778	300
1	11/27/2017	3	34	26244	941	494
1	11/28/2017	3	34	27202	1102	406
1	11/22/2017	3	35	8439	940	140

1	11/23/2017	3	35	12581	1555	277
1	11/24/2017	3	35	8990	1684	392
1	11/25/2017	3	35	11973	1552	427
1	11/26/2017	3	35	13169	2057	427
1	11/27/2017	3	35	11332	1917	532
1	11/28/2017	3	35	10822	1820	524
1	11/22/2017	3	36	5101	1419	141
1	11/23/2017	3	36	12138	1285	224
1	11/24/2017	3	36	13424	1364	271
1	11/25/2017	3	36	15223	1328	379
1	11/26/2017	3	36	10491	1324	220
1	11/27/2017	3	36	13795	1431	499
1	11/28/2017	3	36	14283	1167	289
1	11/22/2017	4	37	9680	841	201
1	11/23/2017	4	37	28498	1073	201
1	11/24/2017	4	37	29671	1720	288
1	11/25/2017	4	37	26108	1501	371
1	11/26/2017	4	37	31575	1426	368
1	11/27/2017	4	37	29934	1633	665
1	11/28/2017	4	37	31401	2189	650
1	11/22/2017	4	38	5507	916	190
1	11/23/2017	4	38	12987	853	138
1	11/24/2017	4	38	17953	843	190
1	11/25/2017	4	38	17331	838	424
1	11/26/2017	4	38	13113	750	155
1	11/27/2017	4	38	17925	1014	232
1	11/28/2017	4	38	22322	882	196
1	11/22/2017	4	39	6333	1172	95
1	11/23/2017	4	39	15877	1233	164
1	11/24/2017	4	39	19541	1183	172
1	11/25/2017	4	39	14657	689	284
1	11/26/2017	4	39	22381	928	261
1	11/27/2017	4	39	21071	1932	350
1	11/28/2017	4	39	33730	1335	416
1	11/22/2017	4	40	5765	422	237
1	11/23/2017	4	40	12899	1023	258
1	11/24/2017	4	40	14603	2018	249
1	11/25/2017	4	40	16891	1671	405
1	11/26/2017	4	40	15265	1989	262
1	11/27/2017	4	40	11874	2411	416
1	11/28/2017	4	40	11220	2158	655
1	11/22/2017	4	41	4275	1052	384
1	11/23/2017	4	41	13078	951	152
1	11/24/2017	4	41	14608	730	134
1	11/25/2017	4	41	11338	905	302
1	11/26/2017	4	41	13619	1128	295
1	11/27/2017	4	41	21348	894	368
1	11/28/2017	4	41	16267	922	515

1	11/22/2017	4	42	7841	886	156
1	11/23/2017	4	42	22447	994	139
1	11/24/2017	4	42	20986	1255	118
1	11/25/2017	4	42	25546	814	202
1	11/26/2017	4	42	25746	1473	243
1	11/27/2017	4	42	39221	1558	332
1	11/28/2017	4	42	33396	1620	401
1	11/22/2017	4	43	5581	793	198
1	11/23/2017	4	43	14250	646	83
1	11/24/2017	4	43	15175	562	219
1	11/25/2017	4	43	27890	482	323
1	11/26/2017	4	43	21880	726	457
1	11/27/2017	4	43	34547	696	277
1	11/28/2017	4	43	33073	731	269
1	11/22/2017	4	44	5723	1010	361
1	11/23/2017	4	44	15120	1554	421
1	11/24/2017	4	44	16911	1397	859
1	11/25/2017	4	44	21638	1306	1316
1	11/26/2017	4	44	19936	1845	722
1	11/27/2017	4	44	21542	1794	1289
1	11/28/2017	4	44	20954	2658	1255
1	11/22/2017	4	45	7301	1243	153
1	11/23/2017	4	45	11274	914	156
1	11/24/2017	4	45	15018	756	197
1	11/25/2017	4	45	15137	712	146
1	11/26/2017	4	45	13866	1012	161
1	11/27/2017	4	45	17633	1075	195
1	11/28/2017	4	45	20417	1115	142
1	11/22/2017	4	46	5688	939	313
1	11/23/2017	4	46	17802	1090	156
1	11/24/2017	4	46	28921	2024	226
1	11/25/2017	4	46	26432	1380	496
1	11/26/2017	4	46	32907	1680	402
1	11/27/2017	4	46	25216	1368	416
1	11/28/2017	4	46	35690	1386	433
1	11/22/2017	4	47	4938	566	144
1	11/23/2017	4	47	19786	827	60
1	11/24/2017	4	47	24274	1411	328
1	11/25/2017	4	47	31887	1051	183
1	11/26/2017	4	47	32589	855	170
1	11/27/2017	4	47	28768	941	155
1	11/28/2017	4	47	34178	1163	176
1	11/22/2017	4	48	10326	518	184
1	11/23/2017	4	48	21982	495	224
1	11/24/2017	4	48	22549	548	373
1	11/25/2017	4	48	25266	587	266
1	11/26/2017	4	48	23187	692	225
1	11/27/2017	4	48	26672	790	372

1	11/28/2017	4	48	27690	570	361
4	12/14/2017	1	1	9841	771	193
4	12/15/2017	1	1	26332	1503	501
4	12/16/2017	1	1	33200	1474	693
4	12/17/2017	1	1	31354	1633	838
4	12/18/2017	1	1	25796	1959	856
4	12/19/2017	1	1	31265	1567	690
4	12/20/2017	1	1	31745	1653	816
4	12/21/2017	1	1	28169	1724	769
4	12/22/2017	1	1	10632	888	269
4	12/14/2017	1	2	9953	578	99
4	12/15/2017	1	2	17409	495	75
4	12/16/2017	1	2	22793	916	150
4	12/17/2017	1	2	33954	678	268
4	12/18/2017	1	2	39984	995	257
4	12/19/2017	1	2	36624	1603	464
4	12/20/2017	1	2	31847	1781	330
4	12/21/2017	1	2	36000	1448	443
4	12/22/2017	1	2	14471	890	193
4	12/14/2017	1	3	8779	1405	194
4	12/15/2017	1	3	27872	1487	620
4	12/16/2017	1	3	28338	1476	720
4	12/17/2017	1	3	31021	1539	754
4	12/18/2017	1	3	26575	1659	595
4	12/19/2017	1	3	29297	1917	828
4	12/20/2017	1	3	24408	2182	1025
4	12/21/2017	1	3	25772	2290	745
4	12/22/2017	1	3	10620	1007	280
4	12/14/2017	1	4	6565	1516	178
4	12/15/2017	1	4	25502	1820	363
4	12/16/2017	1	4	21836	2345	287
4	12/17/2017	1	4	32161	2665	325
4	12/18/2017	1	4	29505	2822	371
4	12/19/2017	1	4	26719	2808	345
4	12/20/2017	1	4	29859	2235	369
4	12/21/2017	1	4	26856	3052	356
4	12/22/2017	1	4	12127	1417	183
4	12/14/2017	1	5	4992	535	95
4	12/15/2017	1	5	10715	713	308
4	12/16/2017	1	5	13895	1097	365
4	12/17/2017	1	5	18581	1388	423
4	12/18/2017	1	5	17279	1908	451
4	12/19/2017	1	5	16927	2224	357
4	12/20/2017	1	5	15893	2285	240
4	12/21/2017	1	5	16428	2622	265
4	12/22/2017	1	5	6949	1360	163
4	12/14/2017	1	6	7962	436	136
4	12/15/2017	1	6	26414	919	484

4	12/16/2017	1	6	31667	1096	721
4	12/17/2017	1	6	32718	1257	661
4	12/18/2017	1	6	32538	1492	770
4	12/19/2017	1	6	39777	1689	839
4	12/20/2017	1	6	35820	1764	552
4	12/21/2017	1	6	35954	1795	677
4	12/22/2017	1	6	14295	1430	358
4	12/14/2017	1	7	12671	506	264
4	12/15/2017	1	7	36576	1125	382
4	12/16/2017	1	7	32085	1844	565
4	12/17/2017	1	7	40211	1884	604
4	12/18/2017	1	7	37209	1873	670
4	12/19/2017	1	7	38164	2026	1006
4	12/20/2017	1	7	34300	1781	626
4	12/21/2017	1	7	29227	2354	1318
4	12/22/2017	1	7	13967	1128	366
4	12/14/2017	1	8	8670	1286	152
4	12/15/2017	1	8	26633	1540	392
4	12/16/2017	1	8	36799	1768	596
4	12/17/2017	1	8	42593	1754	854
4	12/18/2017	1	8	41948	1664	1007
4	12/19/2017	1	8	40934	1944	1199
4	12/20/2017	1	8	29683	1509	1057
4	12/21/2017	1	8	32081	1737	1197
4	12/22/2017	1	8	17985	894	794
4	12/14/2017	1	9	6522	1484	336
4	12/15/2017	1	9	32143	1707	573
4	12/16/2017	1	9	35861	1575	417
4	12/17/2017	1	9	44680	1477	485
4	12/18/2017	1	9	7419	1926	375
4	12/19/2017	1	9	6649	1872	436
4	12/20/2017	1	9	46494	1614	559
4	12/21/2017	1	9	40490	2255	817
4	12/22/2017	1	9	18840	1183	415
4	12/14/2017	1	10	13238	1100	104
4	12/15/2017	1	10	35105	881	488
4	12/16/2017	1	10	34657	1167	865
4	12/17/2017	1	10	32344	1184	623
4	12/18/2017	1	10	30789	1530	489
4	12/19/2017	1	10	33421	1622	691
4	12/20/2017	1	10	34336	1770	689
4	12/21/2017	1	10	31460	2236	602
4	12/22/2017	1	10	16179	933	233
4	12/14/2017	1	11	11970	332	182
4	12/15/2017	1	11	0	0	0
4	12/16/2017	1	11	0	0	0
4	12/17/2017	1	11	0	0	0
4	12/18/2017	1	11	0	0	0

4	12/19/2017	1	11	0	0	0
4	12/20/2017	1	11	0	0	0
4	12/21/2017	1	11	0	0	0
4	12/22/2017	1	11	0	0	0
4	12/14/2017	1	12	9169	460	131
4	12/15/2017	1	12	26382	954	222
4	12/16/2017	1	12	28099	1220	420
4	12/17/2017	1	12	39992	1460	294
4	12/18/2017	1	12	32255	1641	331
4	12/19/2017	1	12	36977	1921	355
4	12/20/2017	1	12	38950	1856	334
4	12/21/2017	1	12	36572	2591	307
4	12/22/2017	1	12	14553	1382	233
4	12/14/2017	2	13	15756	610	105
4	12/15/2017	2	13	32944	2479	453
4	12/16/2017	2	13	34531	3382	326
4	12/17/2017	2	13	29649	4310	415
4	12/18/2017	2	13	31397	4131	463
4	12/19/2017	2	13	33669	4435	598
4	12/20/2017	2	13	35951	3168	649
4	12/21/2017	2	13	31962	3011	650
4	12/22/2017	2	13	12753	1469	320
4	12/14/2017	2	14	10674	711	100
4	12/15/2017	2	14	32253	1339	259
4	12/16/2017	2	14	29086	1502	382
4	12/17/2017	2	14	28014	1222	149
4	12/18/2017	2	14	31964	1468	259
4	12/19/2017	2	14	32663	1549	278
4	12/20/2017	2	14	30472	1795	321
4	12/21/2017	2	14	28702	1731	292
4	12/22/2017	2	14	13220	695	107
4	12/14/2017	2	15	10321	550	154
4	12/15/2017	2	15	38274	1271	583
4	12/16/2017	2	15	39828	1713	650
4	12/17/2017	2	15	40207	1529	775
4	12/18/2017	2	15	39854	1678	1056
4	12/19/2017	2	15	43753	1653	1307
4	12/20/2017	2	15	41244	1579	748
4	12/21/2017	2	15	34809	1837	1538
4	12/22/2017	2	15	16514	912	517
4	12/14/2017	2	16	13643	1235	131
4	12/15/2017	2	16	34671	1804	544
4	12/16/2017	2	16	42885	1819	444
4	12/17/2017	2	16	46456	1806	597
4	12/18/2017	2	16	35519	2226	676
4	12/19/2017	2	16	43253	1863	1082
4	12/20/2017	2	16	42056	1898	751
4	12/21/2017	2	16	38604	2376	895

4	12/22/2017	2	16	20370	1243	346
4	12/14/2017	2	17	14816	760	74
4	12/15/2017	2	17	33853	2042	507
4	12/16/2017	2	17	36048	2086	360
4	12/17/2017	2	17	36701	1553	271
4	12/18/2017	2	17	37947	1688	502
4	12/19/2017	2	17	40269	1850	721
4	12/20/2017	2	17	33550	1880	477
4	12/21/2017	2	17	35349	2241	659
4	12/22/2017	2	17	17663	1342	264
4	12/14/2017	2	18	16222	717	114
4	12/15/2017	2	18	39249	1503	263
4	12/16/2017	2	18	50394	1872	380
4	12/17/2017	2	18	44507	2537	209
4	12/18/2017	2	18	46827	2370	235
4	12/19/2017	2	18	41460	3443	349
4	12/20/2017	2	18	44954	2970	450
4	12/21/2017	2	18	33809	2925	438
4	12/22/2017	2	18	17777	1700	243
4	12/14/2017	2	19	2614	863	107
4	12/15/2017	2	19	30281	897	398
4	12/16/2017	2	19	34926	965	267
4	12/17/2017	2	19	40353	1076	198
4	12/18/2017	2	19	43348	950	241
4	12/19/2017	2	19	35005	1418	365
4	12/20/2017	2	19	40969	1539	336
4	12/21/2017	2	19	26600	2187	428
4	12/22/2017	2	19	17254	720	195
4	12/14/2017	2	20	15659	1859	409
4	12/15/2017	2	20	43129	3758	1513
4	12/16/2017	2	20	45414	3535	1752
4	12/17/2017	2	20	44887	3636	2049
4	12/18/2017	2	20	45408	3455	1998
4	12/19/2017	2	20	42049	4146	1851
4	12/20/2017	2	20	45954	4749	2544
4	12/21/2017	2	20	40606	4235	2046
4	12/22/2017	2	20	18052	1754	1189
4	12/14/2017	2	21	7695	840	120
4	12/15/2017	2	21	28999	1078	206
4	12/16/2017	2	21	27803	1520	316
4	12/17/2017	2	21	23189	1477	256
4	12/18/2017	2	21	29213	1521	315
4	12/19/2017	2	21	25940	1912	325
4	12/20/2017	2	21	23706	2263	236
4	12/21/2017	2	21	21413	2362	467
4	12/22/2017	2	21	10474	908	230
4	12/14/2017	2	22	5839	957	143
4	12/15/2017	2	22	23537	1519	240

4	12/16/2017	2	22	24261	2091	170
4	12/17/2017	2	22	21319	1852	204
4	12/18/2017	2	22	22247	2051	231
4	12/19/2017	2	22	21207	1933	374
4	12/20/2017	2	22	21870	2326	224
4	12/21/2017	2	22	19896	2592	245
4	12/22/2017	2	22	9243	1267	184
4	12/14/2017	2	23	16912	1368	334
4	12/15/2017	2	23	52000	2238	1969
4	12/16/2017	2	23	58961	2502	794
4	12/17/2017	2	23	61366	2054	616
4	12/18/2017	2	23	55904	2747	634
4	12/19/2017	2	23	58973	2766	1026
4	12/20/2017	2	23	50862	3280	714
4	12/21/2017	2	23	48821	3709	993
4	12/22/2017	2	23	22196	1931	383
4	12/14/2017	2	24	12555	887	109
4	12/15/2017	2	24	26737	2049	238
4	12/16/2017	2	24	25126	3513	252
4	12/17/2017	2	24	28726	2043	209
4	12/18/2017	2	24	25834	2215	178
4	12/19/2017	2	24	22942	2342	213
4	12/20/2017	2	24	22283	2494	186
4	12/21/2017	2	24	23912	2598	296
4	12/22/2017	2	24	3004	1092	82
4	12/15/2017	3	25	21853	2023	220
4	12/16/2017	3	25	28780	3349	346
4	12/17/2017	3	25	29276	3806	304
4	12/18/2017	3	25	30871	3757	377
4	12/19/2017	3	25	29738	3393	442
4	12/20/2017	3	25	31026	3148	353
4	12/21/2017	3	25	29897	2649	527
4	12/22/2017	3	25	13707	931	248
4	12/15/2017	3	26	27465	1157	586
4	12/16/2017	3	26	38652	1848	551
4	12/17/2017	3	26	37714	1964	775
4	12/18/2017	3	26	38868	1741	913
4	12/19/2017	3	26	40051	2166	957
4	12/20/2017	3	26	36865	3248	1045
4	12/21/2017	3	26	36309	2904	990
4	12/22/2017	3	26	17504	2255	603
4	12/15/2017	3	27	16945	1808	370
4	12/16/2017	3	27	26435	2092	366
4	12/17/2017	3	27	25725	2246	251
4	12/18/2017	3	27	25933	2550	313
4	12/19/2017	3	27	28025	2765	354
4	12/20/2017	3	27	25932	2610	427
4	12/21/2017	3	27	24259	3234	439

4	12/22/2017	3	27	9853	1506	371
4	12/15/2017	3	28	26210	915	279
4	12/16/2017	3	28	32125	1159	265
4	12/17/2017	3	28	36187	1256	219
4	12/18/2017	3	28	39743	1109	241
4	12/19/2017	3	28	38392	1483	276
4	12/20/2017	3	28	33464	1871	233
4	12/21/2017	3	28	34441	1890	281
4	12/22/2017	3	28	17734	856	256
4	12/15/2017	3	29	20823	929	493
4	12/16/2017	3	29	30526	1218	459
4	12/17/2017	3	29	29667	1501	403
4	12/18/2017	3	29	30291	1884	547
4	12/19/2017	3	29	25843	2273	690
4	12/20/2017	3	29	26505	1695	323
4	12/21/2017	3	29	29517	2053	378
4	12/22/2017	3	29	17669	1300	408
4	12/15/2017	3	30	20111	1203	275
4	12/16/2017	3	30	31204	1540	262
4	12/17/2017	3	30	31406	1914	177
4	12/18/2017	3	30	33748	1750	206
4	12/19/2017	3	30	35011	2131	178
4	12/20/2017	3	30	31227	2332	240
4	12/21/2017	3	30	30788	2884	246
4	12/22/2017	3	30	15810	1351	182
4	12/15/2017	3	31	14244	736	332
4	12/16/2017	3	31	22986	1362	242
4	12/17/2017	3	31	20361	2005	276
4	12/18/2017	3	31	18785	1733	350
4	12/19/2017	3	31	22643	1811	360
4	12/20/2017	3	31	24099	2044	347
4	12/21/2017	3	31	23425	2132	269
4	12/22/2017	3	31	10627	1464	181
4	12/15/2017	3	32	21622	885	289
4	12/16/2017	3	32	33944	1136	285
4	12/17/2017	3	32	32212	1254	278
4	12/18/2017	3	32	32358	1135	381
4	12/19/2017	3	32	34668	1110	402
4	12/20/2017	3	32	33929	1505	353
4	12/21/2017	3	32	30861	1375	316
4	12/22/2017	3	32	13338	643	286
4	12/15/2017	3	33	15254	2341	389
4	12/16/2017	3	33	21746	3276	405
4	12/17/2017	3	33	22838	3184	383
4	12/18/2017	3	33	20474	2897	427
4	12/19/2017	3	33	26165	1968	705
4	12/20/2017	3	33	26190	2323	521
4	12/21/2017	3	33	23907	2561	510

4	12/22/2017	3	33	9282	1411	291
4	12/15/2017	3	34	14659	825	335
4	12/16/2017	3	34	28182	1317	190
4	12/17/2017	3	34	30680	1429	338
4	12/18/2017	3	34	30317	1640	433
4	12/19/2017	3	34	34128	1909	448
4	12/20/2017	3	34	32174	1778	347
4	12/21/2017	3	34	26420	2264	362
4	12/22/2017	3	34	11259	1242	211
4	12/15/2017	3	35	11535	1321	309
4	12/16/2017	3	35	17433	1373	142
4	12/17/2017	3	35	24392	1260	192
4	12/18/2017	3	35	21242	1184	328
4	12/19/2017	3	35	18905	1787	377
4	12/20/2017	3	35	16843	1648	267
4	12/21/2017	3	35	19147	1787	225
4	12/22/2017	3	35	10942	1067	186
4	12/15/2017	3	36	21167	1119	386
4	12/16/2017	3	36	28703	2671	484
4	12/17/2017	3	36	4662	3266	336
4	12/18/2017	3	36	4926	3604	425
4	12/19/2017	3	36	5576	3901	523
4	12/20/2017	3	36	5149	3972	586
4	12/21/2017	3	36	5032	4973	493
4	12/22/2017	3	36	3000	2334	384
4	12/15/2017	4	37	33010	1269	468
4	12/16/2017	4	37	39020	2154	332
4	12/17/2017	4	37	27621	2190	372
4	12/18/2017	4	37	31932	2329	465
4	12/19/2017	4	37	40293	2454	465
4	12/20/2017	4	37	37303	2328	489
4	12/21/2017	4	37	29662	3031	551
4	12/22/2017	4	37	9182	1324	196
4	12/15/2017	4	38	23547	3205	190
4	12/16/2017	4	38	28391	2620	314
4	12/17/2017	4	38	25261	1792	296
4	12/18/2017	4	38	28741	2128	354
4	12/19/2017	4	38	28055	2246	383
4	12/20/2017	4	38	25638	3084	380
4	12/21/2017	4	38	24786	2653	434
4	12/22/2017	4	38	10915	1350	214
4	12/15/2017	4	39	36161	1552	253
4	12/16/2017	4	39	49213	1668	329
4	12/17/2017	4	39	41554	2974	298
4	12/18/2017	4	39	47066	2276	355
4	12/19/2017	4	39	48215	2419	494
4	12/20/2017	4	39	50416	2858	325
4	12/21/2017	4	39	33823	2376	380

4	12/22/2017	4	39	16304	890	136
4	12/15/2017	4	40	29427	1009	363
4	12/16/2017	4	40	38994	2513	293
4	12/17/2017	4	40	34329	2601	261
4	12/18/2017	4	40	30160	2697	330
4	12/19/2017	4	40	32286	2805	234
4	12/20/2017	4	40	36928	3623	347
4	12/21/2017	4	40	34665	3237	430
4	12/22/2017	4	40	17042	1370	181
4	12/15/2017	4	41	22401	1013	397
4	12/16/2017	4	41	25709	1913	341
4	12/17/2017	4	41	25262	2282	221
4	12/18/2017	4	41	31328	1748	364
4	12/19/2017	4	41	33875	1819	323
4	12/20/2017	4	41	33834	2419	249
4	12/21/2017	4	41	29489	2506	353
4	12/22/2017	4	41	12973	842	112
4	12/15/2017	4	42	21313	1656	417
4	12/16/2017	4	42	33050	2596	423
4	12/17/2017	4	42	36635	3270	455
4	12/18/2017	4	42	33588	3920	627
4	12/19/2017	4	42	39310	3407	686
4	12/20/2017	4	42	30014	4202	750
4	12/21/2017	4	42	30348	4688	927
4	12/22/2017	4	42	13708	1681	330
4	12/15/2017	4	43	27517	868	310
4	12/16/2017	4	43	38886	1374	344
4	12/17/2017	4	43	40211	1263	364
4	12/18/2017	4	43	36797	1483	447
4	12/19/2017	4	43	42806	1202	315
4	12/20/2017	4	43	40375	1488	348
4	12/21/2017	4	43	33753	1891	410
4	12/22/2017	4	43	15171	789	195
4	12/15/2017	4	44	19108	1066	577
4	12/16/2017	4	44	29246	1066	1335
4	12/17/2017	4	44	23889	1435	931
4	12/18/2017	4	44	27272	1718	1223
4	12/19/2017	4	44	26321	1383	678
4	12/20/2017	4	44	44545	1498	1020
4	12/21/2017	4	44	33808	1915	1169
4	12/22/2017	4	44	14654	526	803
4	12/15/2017	4	45	20899	1669	405
4	12/16/2017	4	45	28798	2042	375
4	12/17/2017	4	45	25920	1752	316
4	12/18/2017	4	45	26945	1870	250
4	12/19/2017	4	45	31771	1816	342
4	12/20/2017	4	45	28081	1874	333
4	12/21/2017	4	45	27840	1978	372

4	12/22/2017	4	45	10755	1009	267
4	12/15/2017	4	46	16971	1239	368
4	12/16/2017	4	46	33452	1665	199
4	12/17/2017	4	46	30152	1589	232
4	12/18/2017	4	46	38460	2006	350
4	12/19/2017	4	46	35845	1589	343
4	12/20/2017	4	46	38058	2191	307
4	12/21/2017	4	46	32023	1910	337
4	12/22/2017	4	46	17544	754	142
4	12/15/2017	4	47	20224	1469	234
4	12/16/2017	4	47	26943	1873	122
4	12/17/2017	4	47	28800	2877	189
4	12/18/2017	4	47	34911	2082	218
4	12/19/2017	4	47	36712	2739	223
4	12/20/2017	4	47	32555	2959	243
4	12/21/2017	4	47	34338	2791	313
4	12/22/2017	4	47	16334	1084	188
4	12/15/2017	4	48	19290	915	352
4	12/16/2017	4	48	32830	1082	457
4	12/17/2017	4	48	33584	1173	553
4	12/18/2017	4	48	36278	1187	655
4	12/19/2017	4	48	32881	1679	817
4	12/20/2017	4	48	38205	1541	642
4	12/21/2017	4	48	38224	2060	1143
4	12/22/2017	4	48	10047	892	502
7	1/5/2018	1	1	23975	1715	431
7	1/6/2018	1	1	22439	1498	401
7	1/7/2018	1	1	39858	1067	752
7	1/8/2018	1	1	24227	1421	565
7	1/9/2018	1	1	13330	1129	671
7	1/10/2018	1	1	25391	2142	630
7	1/11/2018	1	1	27531	1868	836
7	1/12/2018	1	1	7350	975	311
7	1/5/2018	1	2	36806	1983	507
7	1/6/2018	1	2	38228	1977	692
7	1/7/2018	1	2	44666	1484	932
7	1/8/2018	1	2	36917	1901	852
7	1/9/2018	1	2	41580	1946	598
7	1/10/2018	1	2	44133	2969	777
7	1/11/2018	1	2	49949	2339	810
7	1/12/2018	1	2	22498	1184	581
7	1/5/2018	1	3	21777	2123	519
7	1/6/2018	1	3	23572	2216	423
7	1/7/2018	1	3	34990	2029	1042
7	1/8/2018	1	3	23844	1655	659
7	1/9/2018	1	3	29165	1518	841
7	1/10/2018	1	3	26341	1958	993
7	1/11/2018	1	3	23471	1950	826

7	1/12/2018	1	3	13586	1053	483
7	1/5/2018	1	4	15984	2560	348
7	1/6/2018	1	4	25199	3544	490
7	1/7/2018	1	4	32580	3291	562
7	1/8/2018	1	4	26884	2401	351
7	1/9/2018	1	4	25356	2142	479
7	1/10/2018	1	4	25597	3184	658
7	1/11/2018	1	4	26863	2761	382
7	1/12/2018	1	4	14926	1322	270
7	1/5/2018	1	5	18587	2516	272
7	1/6/2018	1	5	16145	2855	232
7	1/7/2018	1	5	17094	2524	362
7	1/8/2018	1	5	22135	2243	239
7	1/9/2018	1	5	20451	2620	340
7	1/10/2018	1	5	21697	3295	308
7	1/11/2018	1	5	20470	2304	453
7	1/12/2018	1	5	8981	1367	245
7	1/5/2018	1	6	27603	2082	529
7	1/6/2018	1	6	28627	2191	530
7	1/7/2018	1	6	30860	1857	877
7	1/8/2018	1	6	28264	1741	387
7	1/9/2018	1	6	30175	2133	847
7	1/10/2018	1	6	40515	2671	899
7	1/11/2018	1	6	39651	1894	1110
7	1/12/2018	1	6	18530	1061	591
7	1/5/2018	1	7	35513	1249	597
7	1/6/2018	1	7	32813	1810	955
7	1/7/2018	1	7	35680	2079	1048
7	1/8/2018	1	7	31459	1909	648
7	1/9/2018	1	7	35176	2048	1162
7	1/10/2018	1	7	31741	2295	916
7	1/11/2018	1	7	31094	2125	719
7	1/12/2018	1	7	14026	1151	825
7	1/5/2018	1	8	29296	1955	641
7	1/6/2018	1	8	35008	1518	631
7	1/7/2018	1	8	50012	1737	1213
7	1/8/2018	1	8	23614	1512	773
7	1/9/2018	1	8	29893	1098	826
7	1/10/2018	1	8	31563	1875	1108
7	1/11/2018	1	8	33574	1677	1183
7	1/12/2018	1	8	25400	1114	1757
7	1/5/2018	1	9	33927	1760	364
7	1/6/2018	1	9	39625	1590	399
7	1/7/2018	1	9	50875	1629	706
7	1/8/2018	1	9	43804	1741	538
7	1/9/2018	1	9	41828	1650	830
7	1/10/2018	1	9	40087	1939	966
7	1/11/2018	1	9	36492	2203	849

7	1/12/2018	1	9	20377	1175	676
7	1/5/2018	1	10	35477	1719	554
7	1/6/2018	1	10	33356	1670	892
7	1/7/2018	1	10	34647	1877	1120
7	1/8/2018	1	10	35292	1153	686
7	1/9/2018	1	10	45598	1224	1005
7	1/10/2018	1	10	37803	1985	817
7	1/11/2018	1	10	29438	2311	950
7	1/12/2018	1	10	16269	949	993
7	1/5/2018	1	11	32484	2244	447
7	1/6/2018	1	11	34848	2197	521
7	1/7/2018	1	11	33403	1914	608
7	1/8/2018	1	11	41365	1866	544
7	1/9/2018	1	11	34595	2058	493
7	1/10/2018	1	11	32926	3020	749
7	1/11/2018	1	11	30916	2847	642
7	1/12/2018	1	11	11681	1443	243
7	1/5/2018	1	12	41371	1977	339
7	1/6/2018	1	12	37122	2275	368
7	1/7/2018	1	12	36364	2063	479
7	1/8/2018	1	12	35249	2287	431
7	1/9/2018	1	12	32961	1983	476
7	1/10/2018	1	12	30956	2318	435
7	1/11/2018	1	12	27517	2255	399
7	1/12/2018	1	12	15475	1055	401
7	1/5/2018	2	13	38430	1031	253
7	1/6/2018	2	13	27686	1461	312
7	1/7/2018	2	13	43934	1605	505
7	1/8/2018	2	13	38560	1624	243
7	1/9/2018	2	13	33717	2191	584
7	1/10/2018	2	13	32118	2804	312
7	1/11/2018	2	13	25359	2991	419
7	1/12/2018	2	13	12687	1619	197
7	1/5/2018	2	14	26310	1735	311
7	1/6/2018	2	14	25409	1756	176
7	1/7/2018	2	14	34301	1494	208
7	1/8/2018	2	14	29027	1442	257
7	1/9/2018	2	14	32868	1458	226
7	1/10/2018	2	14	25549	2005	491
7	1/11/2018	2	14	23625	2179	221
7	1/12/2018	2	14	15050	1083	258
7	1/5/2018	2	15	31505	2134	469
7	1/6/2018	2	15	33972	2050	966
7	1/7/2018	2	15	33217	1858	1277
7	1/8/2018	2	15	22964	2290	623
7	1/9/2018	2	15	32714	1951	1112
7	1/10/2018	2	15	34027	2200	1203
7	1/11/2018	2	15	29049	2774	1043

7	1/12/2018	2	15	13434	1298	570
7	1/5/2018	2	16	35482	3929	448
7	1/6/2018	2	16	38210	2878	796
7	1/7/2018	2	16	44406	2252	1298
7	1/8/2018	2	16	40080	2916	1024
7	1/9/2018	2	16	35032	2543	1343
7	1/10/2018	2	16	39802	3192	1022
7	1/11/2018	2	16	33008	3431	1195
7	1/12/2018	2	16	24237	1657	699
7	1/5/2018	2	17	30212	2239	424
7	1/6/2018	2	17	30753	2409	505
7	1/7/2018	2	17	33036	2196	1010
7	1/8/2018	2	17	32092	2330	666
7	1/9/2018	2	17	29250	2316	754
7	1/10/2018	2	17	28686	3066	599
7	1/11/2018	2	17	23510	3042	637
7	1/12/2018	2	17	13491	1677	541
7	1/5/2018	2	18	51215	3085	527
7	1/6/2018	2	18	49605	3313	500
7	1/7/2018	2	18	34878	3278	554
7	1/8/2018	2	18	52794	1735	479
7	1/9/2018	2	18	33241	2424	527
7	1/10/2018	2	18	45318	2707	560
7	1/11/2018	2	18	44738	2694	698
7	1/12/2018	2	18	25144	1667	304
7	1/5/2018	2	19	30028	978	455
7	1/6/2018	2	19	16322	1249	300
7	1/7/2018	2	19	22939	1465	386
7	1/8/2018	2	19	29150	1120	347
7	1/9/2018	2	19	23476	1414	347
7	1/10/2018	2	19	15834	2062	418
7	1/11/2018	2	19	18187	2026	534
7	1/12/2018	2	19	9204	1063	158
7	1/5/2018	2	20	37035	3935	1272
7	1/6/2018	2	20	37608	3679	1147
7	1/7/2018	2	20	43120	3140	1086
7	1/8/2018	2	20	35205	4060	1623
7	1/9/2018	2	20	41626	3857	1526
7	1/10/2018	2	20	44101	3800	1620
7	1/11/2018	2	20	37319	3488	2367
7	1/12/2018	2	20	17497	2056	1297
7	1/5/2018	2	21	20720	1923	247
7	1/6/2018	2	21	26023	1673	328
7	1/7/2018	2	21	27264	1710	379
7	1/8/2018	2	21	29475	2009	367
7	1/9/2018	2	21	34895	2048	483
7	1/10/2018	2	21	26266	2464	522
7	1/11/2018	2	21	28953	2168	635

7	1/12/2018	2	21	16556	1001	377
7	1/5/2018	2	22	18211	2617	363
7	1/6/2018	2	22	15990	2567	202
7	1/7/2018	2	22	18599	2244	211
7	1/8/2018	2	22	16811	2231	265
7	1/9/2018	2	22	20102	2591	296
7	1/10/2018	2	22	15413	3233	317
7	1/11/2018	2	22	15671	3471	464
7	1/12/2018	2	22	9248	1891	198
7	1/5/2018	2	23	38070	3181	628
7	1/6/2018	2	23	44380	3098	791
7	1/7/2018	2	23	59938	3128	1165
7	1/8/2018	2	23	42461	2939	683
7	1/9/2018	2	23	47635	3100	1499
7	1/10/2018	2	23	47855	3302	711
7	1/11/2018	2	23	41142	3929	698
7	1/12/2018	2	23	27926	1914	304
7	1/5/2018	2	24	19993	2742	262
7	1/6/2018	2	24	22549	2515	247
7	1/7/2018	2	24	15042	3074	225
7	1/8/2018	2	24	33979	1749	263
7	1/9/2018	2	24	28905	1835	290
7	1/10/2018	2	24	25313	2271	350
7	1/11/2018	2	24	21918	2706	365
7	1/12/2018	2	24	5105	1673	244
7	1/5/2018	3	25	30594	3249	1102
7	1/6/2018	3	25	24590	3136	1180
7	1/7/2018	3	25	40694	2124	1062
7	1/8/2018	3	25	36511	2738	759
7	1/9/2018	3	25	40249	2590	483
7	1/10/2018	3	25	43021	4110	1057
7	1/11/2018	3	25	38021	4142	1589
7	1/12/2018	3	25	14915	2111	872
7	1/5/2018	3	26	31006	3383	1363
7	1/6/2018	3	26	34225	2616	1380
7	1/7/2018	3	26	42485	3242	1758
7	1/8/2018	3	26	36096	3093	1789
7	1/9/2018	3	26	37151	3313	1436
7	1/10/2018	3	26	36288	3561	1337
7	1/11/2018	3	26	30331	3431	1615
7	1/12/2018	3	26	15328	2357	1284
7	1/5/2018	3	27	23750	2973	669
7	1/6/2018	3	27	22134	2352	678
7	1/7/2018	3	27	34641	2087	742
7	1/8/2018	3	27	23977	2245	722
7	1/9/2018	3	27	20460	2031	661
7	1/10/2018	3	27	26466	2318	551
7	1/11/2018	3	27	25533	2854	732

7	1/12/2018	3	27	12477	1901	675
7	1/5/2018	3	28	34783	2038	334
7	1/6/2018	3	28	38575	1578	293
7	1/7/2018	3	28	47800	1700	273
7	1/8/2018	3	28	40075	1826	431
7	1/9/2018	3	28	30175	1933	379
7	1/10/2018	3	28	35574	2095	400
7	1/11/2018	3	28	39574	2157	470
7	1/12/2018	3	28	19062	1201	509
7	1/5/2018	3	29	29398	1938	543
7	1/6/2018	3	29	34306	1888	682
7	1/7/2018	3	29	40742	1688	1076
7	1/8/2018	3	29	30556	1373	529
7	1/9/2018	3	29	24237	1514	637
7	1/10/2018	3	29	32277	2400	461
7	1/11/2018	3	29	41741	2549	803
7	1/12/2018	3	29	12854	1494	877
7	1/5/2018	3	30	19696	2559	262
7	1/6/2018	3	30	17756	2635	282
7	1/7/2018	3	30	27787	2625	262
7	1/8/2018	3	30	18810	2923	268
7	1/9/2018	3	30	22562	2665	324
7	1/10/2018	3	30	22309	2927	235
7	1/11/2018	3	30	21236	2478	303
7	1/12/2018	3	30	12492	1646	168
7	1/5/2018	3	31	20876	1157	371
7	1/6/2018	3	31	21640	1567	430
7	1/7/2018	3	31	29880	1461	355
7	1/8/2018	3	31	25383	1411	316
7	1/9/2018	3	31	23895	1738	431
7	1/10/2018	3	31	27442	2153	423
7	1/11/2018	3	31	26942	1976	548
7	1/12/2018	3	31	13336	1147	248
7	1/5/2018	3	32	38851	1581	286
7	1/6/2018	3	32	35654	1779	604
7	1/7/2018	3	32	37130	1884	337
7	1/8/2018	3	32	37176	1407	373
7	1/9/2018	3	32	28361	1414	359
7	1/10/2018	3	32	36647	2118	371
7	1/11/2018	3	32	35607	1597	476
7	1/12/2018	3	32	12581	936	308
7	1/5/2018	3	33	19317	2672	530
7	1/6/2018	3	33	24193	2256	752
7	1/7/2018	3	33	31645	2591	340
7	1/8/2018	3	33	18825	1835	288
7	1/9/2018	3	33	37279	2303	489
7	1/10/2018	3	33	34960	2465	584
7	1/11/2018	3	33	29613	3087	762

7	1/12/2018	3	33	12966	1715	326
7	1/5/2018	3	34	22831	2402	477
7	1/6/2018	3	34	24521	1841	498
7	1/7/2018	3	34	27384	1732	483
7	1/8/2018	3	34	18380	1994	365
7	1/9/2018	3	34	23285	1729	354
7	1/10/2018	3	34	25810	2383	469
7	1/11/2018	3	34	21659	2459	737
7	1/12/2018	3	34	9992	1577	320
7	1/5/2018	3	35	17204	1905	337
7	1/6/2018	3	35	15263	2248	251
7	1/7/2018	3	35	25365	1692	698
7	1/8/2018	3	35	22046	1627	346
7	1/9/2018	3	35	25693	2045	343
7	1/10/2018	3	35	22130	2125	297
7	1/11/2018	3	35	19760	2197	477
7	1/12/2018	3	35	6959	1229	400
7	1/5/2018	3	36	21677	3482	666
7	1/6/2018	3	36	17265	4432	952
7	1/7/2018	3	36	22732	4037	833
7	1/8/2018	3	36	18736	4250	648
7	1/9/2018	3	36	21459	4302	604
7	1/10/2018	3	36	27351	4169	717
7	1/11/2018	3	36	25112	3960	1005
7	1/12/2018	3	36	12344	2168	591
7	1/5/2018	4	37	0	0	0
7	1/6/2018	4	37	0	0	0
7	1/7/2018	4	37	0	0	0
7	1/8/2018	4	37	0	0	0
7	1/9/2018	4	37	0	0	0
7	1/10/2018	4	37	0	0	0
7	1/11/2018	4	37	0	0	0
7	1/12/2018	4	37	0	0	0
7	1/5/2018	4	38	25618	2822	555
7	1/6/2018	4	38	25669	2615	663
7	1/7/2018	4	38	33914	2420	872
7	1/8/2018	4	38	29846	3674	923
7	1/9/2018	4	38	29211	2337	687
7	1/10/2018	4	38	22547	3450	635
7	1/11/2018	4	38	25721	2935	926
7	1/12/2018	4	38	14694	1389	514
7	1/5/2018	4	39	52168	1565	415
7	1/6/2018	4	39	40646	2134	585
7	1/7/2018	4	39	35943	2014	631
7	1/8/2018	4	39	42034	1444	480
7	1/9/2018	4	39	47891	1740	935
7	1/10/2018	4	39	46428	2259	1102
7	1/11/2018	4	39	45037	2332	1161

7	1/12/2018	4	39	16483	1187	893
7	1/5/2018	4	40	30768	2668	478
7	1/6/2018	4	40	25356	2699	190
7	1/7/2018	4	40	36151	2580	272
7	1/8/2018	4	40	30170	2038	258
7	1/9/2018	4	40	33418	2156	431
7	1/10/2018	4	40	32620	2801	388
7	1/11/2018	4	40	32121	2568	590
7	1/12/2018	4	40	15533	1317	289
7	1/5/2018	4	41	22944	2610	448
7	1/6/2018	4	41	21981	2748	469
7	1/7/2018	4	41	38283	2145	316
7	1/8/2018	4	41	24502	2548	386
7	1/9/2018	4	41	25774	2611	474
7	1/10/2018	4	41	28071	2796	344
7	1/11/2018	4	41	27718	2886	542
7	1/12/2018	4	41	13411	1527	273
7	1/5/2018	4	42	33545	3608	812
7	1/6/2018	4	42	34754	3906	781
7	1/7/2018	4	42	40809	2533	665
7	1/8/2018	4	42	39672	3181	738
7	1/9/2018	4	42	41215	3429	610
7	1/10/2018	4	42	35293	4092	542
7	1/11/2018	4	42	31895	3959	754
7	1/12/2018	4	42	16815	2166	365
7	1/5/2018	4	43	36591	1122	428
7	1/6/2018	4	43	38843	1168	426
7	1/7/2018	4	43	45707	1049	394
7	1/8/2018	4	43	40341	866	364
7	1/9/2018	4	43	39878	1075	374
7	1/10/2018	4	43	39035	1489	662
7	1/11/2018	4	43	35738	1893	591
7	1/12/2018	4	43	17171	706	568
7	1/5/2018	4	44	17169	1830	1097
7	1/6/2018	4	44	40994	1652	1479
7	1/7/2018	4	44	46646	2728	2556
7	1/8/2018	4	44	23782	1666	1035
7	1/9/2018	4	44	21921	1380	1354
7	1/10/2018	4	44	22476	1472	1161
7	1/11/2018	4	44	30068	2120	1880
7	1/12/2018	4	44	14728	1343	1701
7	1/5/2018	4	45	27616	2313	488
7	1/6/2018	4	45	28154	2754	711
7	1/7/2018	4	45	42656	2570	470
7	1/8/2018	4	45	25445	2351	716
7	1/9/2018	4	45	31651	1918	516
7	1/10/2018	4	45	24513	2602	908
7	1/11/2018	4	45	23944	2263	1077

7	1/12/2018	4	45	14246	1260	768
7	1/5/2018	4	46	28133	2469	462
7	1/6/2018	4	46	34047	2066	354
7	1/7/2018	4	46	35613	2247	379
7	1/8/2018	4	46	35221	2780	461
7	1/9/2018	4	46	28819	2779	524
7	1/10/2018	4	46	32074	3141	445
7	1/11/2018	4	46	31189	2566	648
7	1/12/2018	4	46	15340	1204	296
7	1/5/2018	4	47	29677	2973	393
7	1/6/2018	4	47	24176	3928	516
7	1/7/2018	4	47	32587	3250	542
7	1/8/2018	4	47	20888	2980	418
7	1/9/2018	4	47	39170	1982	521
7	1/10/2018	4	47	31351	3022	758
7	1/11/2018	4	47	29264	2683	829
7	1/12/2018	4	47	14617	1475	473
7	1/5/2018	4	48	29911	1832	744
7	1/6/2018	4	48	29450	1872	950
7	1/7/2018	4	48	31153	1878	968
7	1/8/2018	4	48	22273	2324	663
7	1/9/2018	4	48	43106	1921	984
7	1/10/2018	4	48	31690	2424	978
7	1/11/2018	4	48	29665	2484	1166
7	1/12/2018	4	48	14454	1556	1548
12	2/9/2018	1	1	10957	739	269
12	2/10/2018	1	1	22210	1020	376
12	2/11/2018	1	1	30640	951	616
12	2/12/2018	1	1	30455	1033	648
12	2/13/2018	1	1	24193	1101	657
12	2/14/2018	1	1	21890	913	713
12	2/15/2018	1	1	29076	1021	878
12	2/16/2018	1	1	2868	633	297
12	2/9/2018	1	2	12986	881	373
12	2/10/2018	1	2	32186	1767	1163
12	2/11/2018	1	2	37318	2115	1254
12	2/12/2018	1	2	38161	1664	715
12	2/13/2018	1	2	37301	2160	1265
12	2/14/2018	1	2	43878	2062	1327
12	2/15/2018	1	2	40122	2964	1333
12	2/16/2018	1	2	19270	1155	570
12	2/9/2018	1	3	12382	764	208
12	2/10/2018	1	3	35457	1299	756
12	2/11/2018	1	3	29107	1390	697
12	2/12/2018	1	3	33627	1275	518
12	2/13/2018	1	3	29361	1489	585
12	2/14/2018	1	3	36894	902	828
12	2/15/2018	1	3	36797	1030	840

12	2/16/2018	1	3	20726	440	469
12	2/9/2018	1	4	9446	1357	105
12	2/10/2018	1	4	32602	2057	290
12	2/11/2018	1	4	28642	2033	381
12	2/12/2018	1	4	31359	2089	340
12	2/13/2018	1	4	29339	2244	358
12	2/14/2018	1	4	33829	1668	454
12	2/15/2018	1	4	30387	1354	462
12	2/16/2018	1	4	14740	865	158
12	2/9/2018	1	5	7474	232	25
12	2/10/2018	1	5	19851	1162	252
12	2/11/2018	1	5	17766	1263	299
12	2/12/2018	1	5	17818	1320	272
12	2/13/2018	1	5	20585	1506	289
12	2/14/2018	1	5	24613	1317	366
12	2/15/2018	1	5	22767	1043	299
12	2/16/2018	1	5	8927	723	167
12	2/9/2018	1	6	6661	546	165
12	2/10/2018	1	6	30973	1351	649
12	2/11/2018	1	6	32379	1160	556
12	2/12/2018	1	6	31658	1088	486
12	2/13/2018	1	6	33767	1225	723
12	2/14/2018	1	6	34637	981	685
12	2/15/2018	1	6	35975	981	926
12	2/16/2018	1	6	16598	689	305
12	2/9/2018	1	7	11868	176	136
12	2/10/2018	1	7	32433	802	378
12	2/11/2018	1	7	25879	946	517
12	2/12/2018	1	7	28651	813	412
12	2/13/2018	1	7	29722	923	499
12	2/14/2018	1	7	27232	819	617
12	2/15/2018	1	7	32613	847	807
12	2/16/2018	1	7	16486	554	413
12	2/9/2018	1	8	6432	844	179
12	2/10/2018	1	8	33850	1524	418
12	2/11/2018	1	8	25263	1495	709
12	2/12/2018	1	8	40068	954	466
12	2/13/2018	1	8	31324	1262	708
12	2/14/2018	1	8	41476	855	1062
12	2/15/2018	1	8	23787	772	957
12	2/16/2018	1	8	22769	813	1034
12	2/9/2018	1	9	13073	512	78
12	2/10/2018	1	9	39055	1080	302
12	2/11/2018	1	9	36055	1180	528
12	2/12/2018	1	9	37820	1047	358
12	2/13/2018	1	9	40189	1129	457
12	2/14/2018	1	9	43870	908	746
12	2/15/2018	1	9	46885	1058	691

12	2/16/2018	1	9	23419	590	368
12	2/9/2018	1	10	13489	806	222
12	2/10/2018	1	10	37223	1136	366
12	2/11/2018	1	10	33607	1033	451
12	2/12/2018	1	10	33319	1148	465
12	2/13/2018	1	10	35578	1229	561
12	2/14/2018	1	10	39007	856	570
12	2/15/2018	1	10	38622	1018	680
12	2/16/2018	1	10	18588	446	314
12	2/9/2018	1	11	18225	384	224
12	2/10/2018	1	11	38869	1661	210
12	2/11/2018	1	11	39591	1436	533
12	2/12/2018	1	11	39793	1286	417
12	2/13/2018	1	11	39557	1283	391
12	2/14/2018	1	11	39595	1447	548
12	2/15/2018	1	11	41738	1322	626
12	2/16/2018	1	11	13699	659	195
12	2/9/2018	1	12	13169	168	76
12	2/10/2018	1	12	39042	1156	240
12	2/11/2018	1	12	39148	1186	316
12	2/12/2018	1	12	22923	1417	196
12	2/13/2018	1	12	50120	1047	366
12	2/14/2018	1	12	48861	803	306
12	2/15/2018	1	12	51476	808	361
12	2/16/2018	1	12	25807	564	237
12	2/9/2018	2	13	10831	429	234
12	2/10/2018	2	13	21700	1577	388
12	2/11/2018	2	13	25273	1710	513
12	2/12/2018	2	13	26311	1850	502
12	2/13/2018	2	13	25152	1418	521
12	2/14/2018	2	13	31968	1150	738
12	2/15/2018	2	13	28179	1185	668
12	2/16/2018	2	13	12682	923	366
12	2/9/2018	2	14	13301	453	144
12	2/10/2018	2	14	25968	1124	154
12	2/11/2018	2	14	26909	1056	180
12	2/12/2018	2	14	29187	859	212
12	2/13/2018	2	14	27025	987	283
12	2/14/2018	2	14	26663	784	304
12	2/15/2018	2	14	33630	888	188
12	2/16/2018	2	14	12276	468	141
12	2/9/2018	2	15	11800	509	158
12	2/10/2018	2	15	38351	1291	338
12	2/11/2018	2	15	38642	1002	661
12	2/12/2018	2	15	37539	1209	665
12	2/13/2018	2	15	35787	1275	652
12	2/14/2018	2	15	51127	1627	1231
12	2/15/2018	2	15	45129	998	703

12	2/16/2018	2	15	21338	582	355
12	2/9/2018	2	16	18127	938	204
12	2/10/2018	2	16	45181	2050	333
12	2/11/2018	2	16	43452	1851	574
12	2/12/2018	2	16	47831	1366	491
12	2/13/2018	2	16	49107	1482	529
12	2/14/2018	2	16	43202	1116	854
12	2/15/2018	2	16	56304	1345	646
12	2/16/2018	2	16	17819	620	558
12	2/9/2018	2	17	14986	775	172
12	2/10/2018	2	17	25966	2161	369
12	2/11/2018	2	17	23726	1816	576
12	2/12/2018	2	17	26779	1868	544
12	2/13/2018	2	17	27568	1597	475
12	2/14/2018	2	17	30574	1447	1151
12	2/15/2018	2	17	32236	1503	800
12	2/16/2018	2	17	17772	782	710
12	2/9/2018	2	18	21492	672	623
12	2/10/2018	2	18	60161	1407	563
12	2/11/2018	2	18	59972	1117	713
12	2/12/2018	2	18	53413	1426	580
12	2/13/2018	2	18	68588	1446	556
12	2/14/2018	2	18	52037	1226	489
12	2/15/2018	2	18	53686	1242	445
12	2/16/2018	2	18	9259	1258	266
12	2/9/2018	2	19	10081	517	95
12	2/10/2018	2	19	34309	1012	264
12	2/11/2018	2	19	45558	1036	275
12	2/12/2018	2	19	29887	1127	265
12	2/13/2018	2	19	33802	1018	270
12	2/14/2018	2	19	39978	762	297
12	2/15/2018	2	19	31898	978	211
12	2/16/2018	2	19	18424	637	213
12	2/9/2018	2	20	19739	907	330
12	2/10/2018	2	20	52343	1546	975
12	2/11/2018	2	20	53547	1654	1427
12	2/12/2018	2	20	47627	1501	1304
12	2/13/2018	2	20	47534	1555	1095
12	2/14/2018	2	20	20238	1039	227
12	2/15/2018	2	20	15404	811	164
12	2/16/2018	2	20	12168	679	445
12	2/9/2018	2	21	10322	477	283
12	2/10/2018	2	21	10494	700	93
12	2/11/2018	2	21	31051	1194	443
12	2/12/2018	2	21	33214	1040	411
12	2/13/2018	2	21	34847	1123	386
12	2/14/2018	2	21	34940	783	531
12	2/15/2018	2	21	38331	989	570

12	2/16/2018	2	21	14956	679	269
12	2/9/2018	2	22	6803	636	181
12	2/10/2018	2	22	15037	1830	171
12	2/11/2018	2	22	15367	1258	236
12	2/12/2018	2	22	20593	1500	380
12	2/13/2018	2	22	20922	1370	311
12	2/14/2018	2	22	19476	1140	411
12	2/15/2018	2	22	20141	1091	286
12	2/16/2018	2	22	8127	684	222
12	2/9/2018	2	23	17647	738	360
12	2/10/2018	2	23	45879	2318	476
12	2/11/2018	2	23	42604	2030	507
12	2/12/2018	2	23	44362	1591	517
12	2/13/2018	2	23	44990	1781	650
12	2/14/2018	2	23	16673	1143	181
12	2/15/2018	2	23	53833	1671	604
12	2/16/2018	2	23	26572	681	540
12	2/9/2018	2	24	13955	947	142
12	2/10/2018	2	24	24927	1847	191
12	2/11/2018	2	24	26169	1080	200
12	2/12/2018	2	24	24323	1226	271
12	2/13/2018	2	24	26151	1317	254
12	2/14/2018	2	24	22907	909	361
12	2/15/2018	2	24	28412	832	286
12	2/16/2018	2	24	6988	941	156
12	2/9/2018	3	25	16235	796	354
12	2/10/2018	3	25	30181	1432	498
12	2/11/2018	3	25	27594	1135	661
12	2/12/2018	3	25	44793	1702	982
12	2/13/2018	3	25	44225	2457	1118
12	2/14/2018	3	25	44891	2107	1249
12	2/15/2018	3	25	26253	2565	1121
12	2/16/2018	3	25	3343	571	281
12	2/9/2018	3	26	9978	560	171
12	2/10/2018	3	26	28661	1541	732
12	2/11/2018	3	26	32444	1461	993
12	2/12/2018	3	26	28412	1214	1082
12	2/13/2018	3	26	29582	1644	913
12	2/14/2018	3	26	31745	1261	1406
12	2/15/2018	3	26	31047	1229	1316
12	2/16/2018	3	26	14862	868	504
12	2/9/2018	3	27	5716	926	190
12	2/10/2018	3	27	27526	1658	292
12	2/11/2018	3	27	33559	1514	455
12	2/12/2018	3	27	39411	1575	632
12	2/13/2018	3	27	28078	1470	343
12	2/14/2018	3	27	33055	1213	691
12	2/15/2018	3	27	26695	1127	685

12	2/16/2018	3	27	17556	645	388
12	2/9/2018	3	28	13396	406	237
12	2/10/2018	3	28	45051	1081	371
12	2/11/2018	3	28	50622	1021	406
12	2/12/2018	3	28	50727	972	622
12	2/13/2018	3	28	46289	1181	342
12	2/14/2018	3	28	47479	659	474
12	2/15/2018	3	28	40403	968	357
12	2/16/2018	3	28	18800	559	219
12	2/9/2018	3	29	10325	615	444
12	2/10/2018	3	29	14329	1378	369
12	2/11/2018	3	29	22143	1674	560
12	2/12/2018	3	29	11995	1690	393
12	2/13/2018	3	29	28339	1306	601
12	2/14/2018	3	29	17161	820	665
12	2/15/2018	3	29	29142	791	1079
12	2/16/2018	3	29	9154	311	570
12	2/9/2018	3	30	0	0	0
12	2/10/2018	3	30	0	0	0
12	2/11/2018	3	30	0	0	0
12	2/12/2018	3	30	0	0	0
12	2/13/2018	3	30	0	0	0
12	2/14/2018	3	30	0	0	0
12	2/15/2018	3	30	0	0	0
12	2/16/2018	3	30	0	0	0
12	2/9/2018	3	31	8552	437	187
12	2/10/2018	3	31	25313	1200	364
12	2/11/2018	3	31	29266	824	416
12	2/12/2018	3	31	31798	838	504
12	2/13/2018	3	31	23901	919	404
12	2/14/2018	3	31	22374	698	549
12	2/15/2018	3	31	19411	850	491
12	2/16/2018	3	31	8820	424	188
12	2/9/2018	3	32	15912	646	105
12	2/10/2018	3	32	49351	872	321
12	2/11/2018	3	32	39539	760	441
12	2/12/2018	3	32	37470	859	330
12	2/13/2018	3	32	38849	994	363
12	2/14/2018	3	32	35135	873	418
12	2/15/2018	3	32	36313	1059	412
12	2/16/2018	3	32	14926	456	303
12	2/9/2018	3	33	7469	1278	334
12	2/10/2018	3	33	20256	1957	611
12	2/11/2018	3	33	23257	1443	553
12	2/12/2018	3	33	25833	1423	818
12	2/13/2018	3	33	22194	1432	661
12	2/14/2018	3	33	24439	1414	1030
12	2/15/2018	3	33	25449	1453	689

12	2/16/2018	3	33	13439	876	331
12	2/9/2018	3	34	9684	318	106
12	2/10/2018	3	34	9989	940	85
12	2/11/2018	3	34	14025	784	224
12	2/12/2018	3	34	21754	652	261
12	2/13/2018	3	34	12810	623	134
12	2/14/2018	3	34	23679	804	353
12	2/15/2018	3	34	24602	1072	439
12	2/16/2018	3	34	13169	526	154
12	2/9/2018	3	35	8305	354	150
12	2/10/2018	3	35	26797	1058	290
12	2/11/2018	3	35	26155	934	274
12	2/12/2018	3	35	33495	1020	361
12	2/13/2018	3	35	29213	925	345
12	2/14/2018	3	35	38399	857	465
12	2/15/2018	3	35	40637	1042	360
12	2/16/2018	3	35	19601	563	234
12	2/9/2018	3	36	9682	335	153
12	2/10/2018	3	36	35152	1472	561
12	2/11/2018	3	36	37104	1532	599
12	2/12/2018	3	36	36901	1589	612
12	2/13/2018	3	36	37945	1838	498
12	2/14/2018	3	36	35163	1529	647
12	2/15/2018	3	36	31831	1564	437
12	2/16/2018	3	36	16855	922	350
12	2/9/2018	4	37	7535	701	268
12	2/10/2018	4	37	28103	1011	451
12	2/11/2018	4	37	35544	1107	476
12	2/12/2018	4	37	28403	1160	450
12	2/13/2018	4	37	49089	1117	646
12	2/14/2018	4	37	46088	1472	837
12	2/15/2018	4	37	44302	1312	561
12	2/16/2018	4	37	16290	629	281
12	2/9/2018	4	38	10902	1202	132
12	2/10/2018	4	38	23268	2066	469
12	2/11/2018	4	38	28568	1497	512
12	2/12/2018	4	38	29864	1374	477
12	2/13/2018	4	38	29453	1155	523
12	2/14/2018	4	38	31675	1076	576
12	2/15/2018	4	38	31332	1578	576
12	2/16/2018	4	38	11088	645	314
12	2/9/2018	4	39	16528	741	215
12	2/10/2018	4	39	32047	1025	565
12	2/11/2018	4	39	29105	1052	671
12	2/12/2018	4	39	38787	890	520
12	2/13/2018	4	39	40940	1018	701
12	2/14/2018	4	39	36017	1221	933
12	2/15/2018	4	39	36704	1441	713

12	2/16/2018	4	39	10216	780	508
12	2/9/2018	4	40	13029	468	223
12	2/10/2018	4	40	33921	1694	286
12	2/11/2018	4	40	33426	1772	354
12	2/12/2018	4	40	34109	1397	379
12	2/13/2018	4	40	38171	1537	330
12	2/14/2018	4	40	39633	1517	352
12	2/15/2018	4	40	33117	1673	302
12	2/16/2018	4	40	12912	754	174
12	2/9/2018	4	41	9019	628	455
12	2/10/2018	4	41	28733	1271	324
12	2/11/2018	4	41	26761	1533	260
12	2/12/2018	4	41	27660	1243	268
12	2/13/2018	4	41	26181	1282	263
12	2/14/2018	4	41	28376	1100	313
12	2/15/2018	4	41	24212	1317	280
12	2/16/2018	4	41	9018	589	193
12	2/9/2018	4	42	14821	807	208
12	2/10/2018	4	42	34791	2275	571
12	2/11/2018	4	42	38855	2232	577
12	2/12/2018	4	42	42522	1955	600
12	2/13/2018	4	42	45673	1870	523
12	2/14/2018	4	42	40273	1892	656
12	2/15/2018	4	42	43276	2087	764
12	2/16/2018	4	42	15814	1083	204
12	2/9/2018	4	43	8383	187	81
12	2/10/2018	4	43	37260	885	411
12	2/11/2018	4	43	40575	1341	345
12	2/12/2018	4	43	39022	867	419
12	2/13/2018	4	43	44502	927	462
12	2/14/2018	4	43	35234	1013	475
12	2/15/2018	4	43	42485	959	355
12	2/16/2018	4	43	16900	636	301
12	2/9/2018	4	44	5063	710	421
12	2/10/2018	4	44	19718	854	1238
12	2/11/2018	4	44	27627	944	949
12	2/12/2018	4	44	37926	1173	1222
12	2/13/2018	4	44	31359	702	702
12	2/14/2018	4	44	41214	1400	878
12	2/15/2018	4	44	36779	1447	826
12	2/16/2018	4	44	32710	833	1189
12	2/9/2018	4	45	7138	1275	272
12	2/10/2018	4	45	20958	2029	533
12	2/11/2018	4	45	26005	2914	629
12	2/12/2018	4	45	25848	1838	739
12	2/13/2018	4	45	25142	1365	605
12	2/14/2018	4	45	27240	2130	794
12	2/15/2018	4	45	27543	1332	679

12	2/16/2018	4	45	12179	900	485
12	2/9/2018	4	46	12092	548	239
12	2/10/2018	4	46	31546	1768	254
12	2/11/2018	4	46	30527	1445	274
12	2/12/2018	4	46	29814	1423	332
12	2/13/2018	4	46	36303	1284	282
12	2/14/2018	4	46	42692	978	337
12	2/15/2018	4	46	32465	1431	292
12	2/16/2018	4	46	15957	566	148
12	2/9/2018	4	47	10127	613	230
12	2/10/2018	4	47	31205	1401	343
12	2/11/2018	4	47	31539	1753	441
12	2/12/2018	4	47	36004	1360	466
12	2/13/2018	4	47	39803	1192	546
12	2/14/2018	4	47	48769	1261	753
12	2/15/2018	4	47	43558	1317	660
12	2/16/2018	4	47	11982	958	345
12	2/9/2018	4	48	12870	718	614
12	2/10/2018	4	48	31349	1409	2147
12	2/11/2018	4	48	36446	1353	2583
12	2/12/2018	4	48	38010	1202	1852
12	2/13/2018	4	48	32579	1143	1798
12	2/14/2018	4	48	26158	961	1620
12	2/15/2018	4	48	32286	969	2048
12	2/16/2018	4	48	10153	696	559
19	3/29/2018	1	1	16066	491	229
19	3/30/2018	1	1	19208	973	341
19	3/31/2018	1	1	28947	903	404
19	4/1/2018	1	1	21620	1114	436
19	4/2/2018	1	1	22805	960	344
19	4/3/2018	1	1	31046	790	360
19	4/4/2018	1	1	24938	907	512
19	4/5/2018	1	1	4623	631	128
19	3/29/2018	1	2	23654	432	127
19	3/30/2018	1	2	49471	645	250
19	3/31/2018	1	2	42023	923	563
19	4/1/2018	1	2	51381	818	206
19	4/2/2018	1	2	37952	1165	526
19	4/3/2018	1	2	37641	1139	395
19	4/4/2018	1	2	32340	1088	528
19	4/5/2018	1	2	6363	697	145
19	3/29/2018	1	3	16581	872	277
19	3/30/2018	1	3	27682	1206	484
19	3/31/2018	1	3	42930	820	522
19	4/1/2018	1	3	31440	1698	702
19	4/2/2018	1	3	34157	1260	730
19	4/3/2018	1	3	31569	1232	554
19	4/4/2018	1	3	32795	1131	584

19	4/5/2018	1	3	13724	593	236
19	3/29/2018	1	4	11710	929	145
19	3/30/2018	1	4	25429	994	160
19	3/31/2018	1	4	29490	1374	249
19	4/1/2018	1	4	28250	1716	234
19	4/2/2018	1	4	24856	1916	323
19	4/3/2018	1	4	25784	1490	174
19	4/4/2018	1	4	24714	1322	319
19	4/5/2018	1	4	8622	991	108
19	3/29/2018	1	5	8686	227	43
19	3/30/2018	1	5	19383	640	167
19	3/31/2018	1	5	15816	1035	380
19	4/1/2018	1	5	21941	895	156
19	4/2/2018	1	5	17957	1224	289
19	4/3/2018	1	5	15907	1073	265
19	4/4/2018	1	5	17362	877	264
19	4/5/2018	1	5	2784	576	160
19	3/29/2018	1	6	9168	450	96
19	3/30/2018	1	6	19111	1036	190
19	3/31/2018	1	6	35483	1014	273
19	4/1/2018	1	6	22247	1483	259
19	4/2/2018	1	6	32911	1231	315
19	4/3/2018	1	6	24365	1234	304
19	4/4/2018	1	6	35311	841	316
19	4/5/2018	1	6	10751	752	134
19	3/29/2018	1	7	18199	181	111
19	3/30/2018	1	7	34016	473	198
19	3/31/2018	1	7	34026	761	405
19	4/1/2018	1	7	44634	725	312
19	4/2/2018	1	7	37860	624	417
19	4/3/2018	1	7	37275	679	314
19	4/4/2018	1	7	37275	683	348
19	4/5/2018	1	7	8893	381	131
19	3/29/2018	1	8	15584	677	359
19	3/30/2018	1	8	30120	690	593
19	3/31/2018	1	8	48337	1002	536
19	4/1/2018	1	8	40050	966	554
19	4/2/2018	1	8	42708	1140	884
19	4/3/2018	1	8	36865	924	730
19	4/4/2018	1	8	30557	764	860
19	4/5/2018	1	8	28962	467	428
19	3/29/2018	1	9	21018	262	110
19	3/30/2018	1	9	34601	978	349
19	3/31/2018	1	9	40568	1166	539
19	4/1/2018	1	9	48925	856	330
19	4/2/2018	1	9	44173	1237	816
19	4/3/2018	1	9	40847	1184	673
19	4/4/2018	1	9	46008	906	654

19	4/5/2018	1	9	11276	615	289
19	3/29/2018	1	10	17592	563	125
19	3/30/2018	1	10	37944	796	294
19	3/31/2018	1	10	48427	939	474
19	4/1/2018	1	10	36524	1316	425
19	4/2/2018	1	10	40799	1102	748
19	4/3/2018	1	10	36592	1062	388
19	4/4/2018	1	10	41058	775	459
19	4/5/2018	1	10	13741	494	228
19	3/29/2018	1	11	22331	703	165
19	3/30/2018	1	11	37668	2478	315
19	3/31/2018	1	11	35530	2167	339
19	4/1/2018	1	11	38952	2360	395
19	4/2/2018	1	11	43371	1931	334
19	4/3/2018	1	11	44958	2518	438
19	4/4/2018	1	11	47841	2257	605
19	4/5/2018	1	11	12747	1234	117
19	3/29/2018	1	12	25124	382	128
19	3/30/2018	1	12	45757	806	357
19	3/31/2018	1	12	63793	844	289
19	4/1/2018	1	12	49386	934	272
19	4/2/2018	1	12	53203	1007	331
19	4/3/2018	1	12	52881	1041	454
19	4/4/2018	1	12	54822	670	332
19	4/5/2018	1	12	14062	722	193
19	3/29/2018	2	13	14125	270	56
19	3/30/2018	2	13	27392	1165	216
19	3/31/2018	2	13	34931	1131	315
19	4/1/2018	2	13	30973	1154	267
19	4/2/2018	2	13	39654	850	402
19	4/3/2018	2	13	41970	971	485
19	4/4/2018	2	13	37522	935	457
19	4/5/2018	2	13	9149	758	163
19	3/29/2018	2	14	16456	449	99
19	3/30/2018	2	14	26484	870	177
19	3/31/2018	2	14	36833	1006	216
19	4/1/2018	2	14	27921	795	158
19	4/2/2018	2	14	32340	709	282
19	4/3/2018	2	14	28425	849	210
19	4/4/2018	2	14	32956	703	263
19	4/5/2018	2	14	2934	607	101
19	3/29/2018	2	15	19786	474	175
19	3/30/2018	2	15	37660	1139	457
19	3/31/2018	2	15	54789	949	670
19	4/1/2018	2	15	48487	1176	420
19	4/2/2018	2	15	52249	965	568
19	4/3/2018	2	15	51596	1047	556
19	4/4/2018	2	15	52477	874	743

19	4/5/2018	2	15	14766	676	297
19	3/29/2018	2	16	28463	885	147
19	3/30/2018	2	16	44227	1455	353
19	3/31/2018	2	16	49710	1281	442
19	4/1/2018	2	16	56915	1418	379
19	4/2/2018	2	16	52984	1126	454
19	4/3/2018	2	16	45636	1078	557
19	4/4/2018	2	16	55316	1015	521
19	4/5/2018	2	16	8529	892	225
19	3/29/2018	2	17	10829	401	130
19	3/30/2018	2	17	25493	1282	207
19	3/31/2018	2	17	28727	1280	294
19	4/1/2018	2	17	17184	1878	229
19	4/2/2018	2	17	23462	2008	308
19	4/3/2018	2	17	22937	1328	293
19	4/4/2018	2	17	22972	1100	345
19	4/5/2018	2	17	8439	685	239
19	3/29/2018	2	18	36142	480	197
19	3/30/2018	2	18	56500	1599	314
19	3/31/2018	2	18	30134	2193	573
19	4/1/2018	2	18	85454	1331	312
19	4/2/2018	2	18	50597	2179	533
19	4/3/2018	2	18	49328	1690	455
19	4/4/2018	2	18	56013	1590	430
19	4/5/2018	2	18	2701	1153	112
19	3/29/2018	2	19	15967	569	104
19	3/30/2018	2	19	26033	929	193
19	3/31/2018	2	19	24055	851	234
19	4/1/2018	2	19	8602	643	88
19	4/2/2018	2	19	28708	847	220
19	4/3/2018	2	19	35475	766	393
19	4/4/2018	2	19	40440	832	283
19	4/5/2018	2	19	4211	647	115
19	3/29/2018	2	20	25653	699	284
19	3/30/2018	2	20	43393	1504	289
19	3/31/2018	2	20	42361	2414	778
19	4/1/2018	2	20	44456	1271	346
19	4/2/2018	2	20	48313	1789	607
19	4/3/2018	2	20	49552	1651	732
19	4/4/2018	2	20	52481	1500	603
19	4/5/2018	2	20	5562	1103	137
19	3/29/2018	2	21	19062	430	154
19	3/30/2018	2	21	34508	820	260
19	3/31/2018	2	21	35223	970	393
19	4/1/2018	2	21	38202	837	255
19	4/2/2018	2	21	40285	889	311
19	4/3/2018	2	21	37063	834	301
19	4/4/2018	2	21	10787	215	47

19	4/5/2018	2	21	10391	276	128
19	3/29/2018	2	22	10941	457	157
19	3/30/2018	2	22	17340	905	138
19	3/31/2018	2	22	20649	760	240
19	4/1/2018	2	22	19990	1053	193
19	4/2/2018	2	22	19260	1020	222
19	4/3/2018	2	22	22744	892	228
19	4/4/2018	2	22	20966	786	231
19	4/5/2018	2	22	3340	627	90
19	3/29/2018	2	23	27892	446	269
19	3/30/2018	2	23	40257	1191	420
19	3/31/2018	2	23	53861	887	658
19	4/1/2018	2	23	55804	1310	620
19	4/2/2018	2	23	65205	913	536
19	4/3/2018	2	23	57095	1059	453
19	4/4/2018	2	23	58580	1169	447
19	4/5/2018	2	23	9823	891	153
19	3/29/2018	2	24	16413	640	84
19	3/30/2018	2	24	21743	1233	128
19	3/31/2018	2	24	20146	1112	182
19	4/1/2018	2	24	25770	1019	147
19	4/2/2018	2	24	25426	1415	222
19	4/3/2018	2	24	26367	910	248
19	4/4/2018	2	24	30107	941	260
19	4/5/2018	2	24	5314	892	91
19	3/29/2018	3	25	25495	1256	160
19	3/30/2018	3	25	33059	2638	324
19	3/31/2018	3	25	31922	3695	561
19	4/1/2018	3	25	43029	3759	772
19	4/2/2018	3	25	33906	3588	1066
19	4/3/2018	3	25	31570	3614	818
19	4/4/2018	3	25	37024	3547	884
19	4/5/2018	3	25	6682	1459	204
19	3/29/2018	3	26	15806	665	153
19	3/30/2018	3	26	27783	2586	505
19	3/31/2018	3	26	37835	2206	594
19	4/1/2018	3	26	32245	2850	574
19	4/2/2018	3	26	39661	2607	774
19	4/3/2018	3	26	35885	2298	695
19	4/4/2018	3	26	38327	1978	973
19	4/5/2018	3	26	11722	1416	351
19	3/29/2018	3	27	11150	750	155
19	3/30/2018	3	27	22117	1845	227
19	3/31/2018	3	27	29511	1963	350
19	4/1/2018	3	27	26407	2413	305
19	4/2/2018	3	27	31588	2249	479
19	4/3/2018	3	27	28677	2247	563
19	4/4/2018	3	27	30024	1958	353

19	4/5/2018	3	27	11195	1265	219
19	3/29/2018	3	28	25407	359	188
19	3/30/2018	3	28	39763	1363	320
19	3/31/2018	3	28	38237	1620	440
19	4/1/2018	3	28	51542	1501	277
19	4/2/2018	3	28	41131	2144	472
19	4/3/2018	3	28	40204	1997	420
19	4/4/2018	3	28	44510	1825	438
19	4/5/2018	3	28	5906	1454	265
19	3/29/2018	3	29	3240	247	166
19	3/30/2018	3	29	2903	379	147
19	3/31/2018	3	29	3326	542	216
19	4/1/2018	3	29	2136	529	152
19	4/2/2018	3	29	3603	646	294
19	4/3/2018	3	29	5364	1269	467
19	4/4/2018	3	29	3272	691	298
19	4/5/2018	3	29	204	364	132
19	3/29/2018	3	30	0	0	0
19	3/30/2018	3	30	0	0	0
19	3/31/2018	3	30	0	0	0
19	4/1/2018	3	30	0	0	0
19	4/2/2018	3	30	0	0	0
19	4/3/2018	3	30	0	0	0
19	4/4/2018	3	30	0	0	0
19	4/5/2018	3	30	0	0	0
19	3/29/2018	3	31	7231	337	55
19	3/30/2018	3	31	13390	857	156
19	3/31/2018	3	31	14657	1524	173
19	4/1/2018	3	31	14525	1313	172
19	4/2/2018	3	31	14061	1396	276
19	4/3/2018	3	31	18252	1135	293
19	4/4/2018	3	31	19491	1189	268
19	4/5/2018	3	31	2109	874	143
19	3/29/2018	3	32	18123	409	65
19	3/30/2018	3	32	28752	923	181
19	3/31/2018	3	32	30239	1127	341
19	4/1/2018	3	32	36322	1147	288
19	4/2/2018	3	32	40573	1186	407
19	4/3/2018	3	32	40509	1181	373
19	4/4/2018	3	32	40818	1036	343
19	4/5/2018	3	32	7384	1038	169
19	3/29/2018	3	33	11122	1273	185
19	3/30/2018	3	33	26585	2576	334
19	3/31/2018	3	33	24010	2855	380
19	4/1/2018	3	33	28948	2514	256
19	4/2/2018	3	33	23411	2194	360
19	4/3/2018	3	33	26241	2253	408
19	4/4/2018	3	33	30861	1919	344

19	4/5/2018	3	33	5664	1210	215
19	3/29/2018	3	34	9339	502	192
19	3/30/2018	3	34	24293	1400	289
19	3/31/2018	3	34	37107	1298	350
19	4/1/2018	3	34	17166	1523	334
19	4/2/2018	3	34	31019	1517	461
19	4/3/2018	3	34	29245	1661	687
19	4/4/2018	3	34	29860	1455	511
19	4/5/2018	3	34	13758	847	202
19	3/29/2018	3	35	13575	409	141
19	3/30/2018	3	35	22314	1448	247
19	3/31/2018	3	35	28120	1340	333
19	4/1/2018	3	35	24782	1522	251
19	4/2/2018	3	35	31103	1500	344
19	4/3/2018	3	35	28301	1259	258
19	4/4/2018	3	35	31668	1260	396
19	4/5/2018	3	35	5180	1088	233
19	3/29/2018	3	36	11040	492	147
19	3/30/2018	3	36	18725	1883	369
19	3/31/2018	3	36	33650	1959	716
19	4/1/2018	3	36	21003	3055	413
19	4/2/2018	3	36	21632	3087	647
19	4/3/2018	3	36	21341	2589	603
19	4/4/2018	3	36	24286	2522	579
19	4/5/2018	3	36	8353	1460	434
19	3/29/2018	4	37	21817	1037	378
19	3/30/2018	4	37	48834	1949	489
19	3/31/2018	4	37	41152	2420	588
19	4/1/2018	4	37	49666	1777	800
19	4/2/2018	4	37	42237	2224	616
19	4/3/2018	4	37	44465	2563	934
19	4/4/2018	4	37	44023	2318	1188
19	4/5/2018	4	37	8882	1241	355
19	3/29/2018	4	38	13623	1404	147
19	3/30/2018	4	38	27273	1951	421
19	3/31/2018	4	38	36809	2365	443
19	4/1/2018	4	38	34344	2301	546
19	4/2/2018	4	38	36403	2160	450
19	4/3/2018	4	38	33620	2470	462
19	4/4/2018	4	38	33711	1857	419
19	4/5/2018	4	38	6543	913	176
19	3/29/2018	4	39	16359	680	112
19	3/30/2018	4	39	27301	2112	219
19	3/31/2018	4	39	27500	1788	408
19	4/1/2018	4	39	32530	2287	314
19	4/2/2018	4	39	32972	1675	444
19	4/3/2018	4	39	31641	1657	521
19	4/4/2018	4	39	33915	1469	500

19	4/5/2018	4	39	7494	1054	321
19	3/29/2018	4	40	23627	1085	229
19	3/30/2018	4	40	44288	1637	389
19	3/31/2018	4	40	50577	2133	519
19	4/1/2018	4	40	50656	1805	352
19	4/2/2018	4	40	54546	1633	312
19	4/3/2018	4	40	52994	1852	357
19	4/4/2018	4	40	53394	1979	838
19	4/5/2018	4	40	16381	922	185
19	3/29/2018	4	41	9794	1142	425
19	3/30/2018	4	41	20605	2698	519
19	3/31/2018	4	41	21385	2998	440
19	4/1/2018	4	41	25950	3046	542
19	4/2/2018	4	41	21573	2145	513
19	4/3/2018	4	41	28128	2193	386
19	4/4/2018	4	41	30089	2180	498
19	4/5/2018	4	41	10944	726	241
19	3/29/2018	4	42	22331	703	165
19	3/30/2018	4	42	37668	2478	315
19	3/31/2018	4	42	35531	2167	339
19	4/1/2018	4	42	38951	2360	395
19	4/2/2018	4	42	43370	1931	334
19	4/3/2018	4	42	44958	2518	438
19	4/4/2018	4	42	47842	2257	605
19	4/5/2018	4	42	12747	1234	117
19	3/29/2018	4	43	22748	401	181
19	3/30/2018	4	43	42522	1232	328
19	3/31/2018	4	43	36370	1364	453
19	4/1/2018	4	43	46177	1049	453
19	4/2/2018	4	43	40815	1165	446
19	4/3/2018	4	43	40171	1292	502
19	4/4/2018	4	43	38466	1094	726
19	4/5/2018	4	43	10934	684	236
19	3/29/2018	4	44	12669	860	862
19	3/30/2018	4	44	20417	1546	716
19	3/31/2018	4	44	27179	1763	1610
19	4/1/2018	4	44	33281	1677	1872
19	4/2/2018	4	44	36915	1769	2133
19	4/3/2018	4	44	30333	1949	2017
19	4/4/2018	4	44	35757	1814	2897
19	4/5/2018	4	44	11573	729	1021
19	3/29/2018	4	45	5883	1209	165
19	3/30/2018	4	45	21648	3440	380
19	3/31/2018	4	45	27441	2457	496
19	4/1/2018	4	45	21925	3798	531
19	4/2/2018	4	45	23105	2005	608
19	4/3/2018	4	45	25615	2217	602
19	4/4/2018	4	45	27266	2275	732

19	4/5/2018	4	45	8575	1322	365
19	3/29/2018	4	46	15028	379	223
19	3/30/2018	4	46	30447	1936	238
19	3/31/2018	4	46	35023	1776	301
19	4/1/2018	4	46	37753	1924	362
19	4/2/2018	4	46	36842	1699	382
19	4/3/2018	4	46	38434	1703	464
19	4/4/2018	4	46	39539	1731	403
19	4/5/2018	4	46	9390	813	195
19	3/29/2018	4	47	7734	785	232
19	3/30/2018	4	47	30864	1733	482
19	3/31/2018	4	47	7489	1680	491
19	4/1/2018	4	47	23484	2311	697
19	4/2/2018	4	47	25487	1751	624
19	4/3/2018	4	47	30830	2218	857
19	4/4/2018	4	47	41726	2427	1538
19	4/5/2018	4	47	13178	1145	432
19	3/29/2018	4	48	20650	823	615
19	3/30/2018	4	48	37489	1825	979
19	3/31/2018	4	48	43251	1449	1454
19	4/1/2018	4	48	44307	1796	1732
19	4/2/2018	4	48	59770	1914	2115
19	4/3/2018	4	48	45883	1984	2257
19	4/4/2018	4	48	42925	1754	3144
19	4/5/2018	4	48	15257	1289	1018
