# Supplementary material for: Parasitic mites alter chicken behaviour and negatively impact animal welfare
Source: Sci Rep. 2020 May 19;10:8236. doi: 10.1038/s41598-020-65021-0 (PMC7237419; doi:10.1038/s41598-020-65021-0)
Supplement: Supplementary file 2 — Supplementary information2. [file 41598_2020_65021_MOESM2_ESM.pdf]

Supplementary Table 2 to:

Parasitic mites alter chicken behaviour and negatively impact animal welfare

Amy C. Murillo*[1], Alireza Abdoli[2], Richard A. Blatchford[3], Eamonn J. Keogh[2], and Alec C. Gerry[1]

*Corresponding author: Amy Murillo, Dept. of Entomology, Univ. of California, Riverside, CA, 92521, USA +1 (951) 827-5741, amy.murillo@ucr.edu

[1] Department of Entomology, University of California, Riverside, CA

[2] Department of Computer Science & Engineering, University of California, Riverside, CA

[3] Department of Animal Science, Center for Animal Welfare, University of California, Davis, CA

Date (2017-2018)	Week	Flock	Chicken	Eye	Nose	Comb	Beak	Feather	Foot	Toe	Keel	Soiled	Skin	NFM score
22-Nov	1	1	1	0	0	0	1	0	0	0	0	0	0	0
22-Nov	1	1	2	0	0	0	1	0	0	0	0	0	0	0
22-Nov	1	1	3	0	0	0	1	0	0	0	0	0	0	0
22-Nov	1	1	4	0	0	0	1	0	0	0	0	0	0	0
22-Nov	1	1	5	0	0	0	2	0	0	0	0	0	0	0
22-Nov	1	1	6	0	0	0	2	0	0	0	0	0	0	0
22-Nov	1	1	7	0	0	0	1	0	0	0	0	0	0	0
22-Nov	1	1	8	0	0	0	1	0	0	0	0	0	0	0
22-Nov	1	1	9	0	0	0	1	0	0	0	0	0	0	0
22-Nov	1	1	10	0	0	0	1	0	0	0	0	0	0	0
22-Nov	1	1	11	0	0	0	1	0	0	0	0	0	0	0
22-Nov	1	1	12	0	0	0	1	0	0	0	0	0	0	0
22-Nov	1	2	13	0	0	0	1	0	0	0	0	0	0	0
22-Nov	1	2	14	0	0	0	1	0	0	0	0	0	0	0
22-Nov	1	2	15	0	0	0	1	0	0	0	0	0	0	0
22-Nov	1	2	16	0	0	0	1	0	0	0	0	0	0	0
22-Nov	1	2	17	0	0	0	1	0	0	0	1	0	0	0
22-Nov	1	2	18	0	0	0	1	0	0	0	0	0	0	0
22-Nov	1	2	19	0	0	0	1	0	0	0	0	0	0	0
22-Nov	1	2	20	0	0	0	1	0	0	0	0	0	0	0
22-Nov	1	2	21	0	0	0	2	0	0	0	0	0	0	0
22-Nov	1	2	22	0	0	0	1	0	0	0	0	0	0	0
22-Nov	1	2	23	0	0	0	1	0	0	0	0	0	0	0
22-Nov	1	2	24	0	0	0	2	0	0	0	0	0	0	0
22-Nov	1	3	25	0	0	0	1	0	0	0	0	0	0	0
22-Nov	1	3	26	0	0	0	1	0	0	0	0	0	0	0
22-Nov	1	3	27	0	0	0	1	0	0	0	0	0	0	0

22-Nov	1	3	28	0	0	0	1	0	0	0	0	0	0	0
22-Nov	1	3	29	0	0	0	1	0	0	0	0	0	0	0
22-Nov	1	3	30	0	0	0	1	0	0	1	0	1	0	0
22-Nov	1	3	31	0	0	0	1	0	0	0	0	0	0	0
22-Nov	1	3	32	0	0	0	1	0	0	0	0	0	0	0
22-Nov	1	3	33	0	0	0	1	0	0	0	0	0	0	0
22-Nov	1	3	34	0	0	0	1	0	0	0	0	0	0	0
22-Nov	1	3	35	0	0	0	1	0	0	0	0	1	0	0
22-Nov	1	3	36	0	0	0	1	0	0	0	0	0	0	0
22-Nov	1	4	37	0	0	0	1	0	0	0	0	0	0	0
22-Nov	1	4	38	0	0	0	1	0	0	0	0	0	0	0
22-Nov	1	4	39	0	0	0	1	0	0	0	0	0	0	0
22-Nov	1	4	40	0	0	0	1	0	0	0	0	0	0	0
22-Nov	1	4	41	0	0	0	1	0	0	0	0	0	0	0
22-Nov	1	4	42	0	0	0	1	0	0	0	0	0	0	0
22-Nov	1	4	43	0	0	0	1	0	0	0	0	0	0	0
22-Nov	1	4	44	0	0	0	1	0	0	0	0	0	0	0
22-Nov	1	4	45	0	0	0	1	0	0	0	0	0	0	0
22-Nov	1	4	46	0	0	0	1	0	0	0	0	0	0	0
22-Nov	1	4	47	0	0	0	1	0	0	0	0	0	0	0
22-Nov	1	4	48	0	0	0	1	0	0	0	0	0	0	0
14-Dec	4	1	1	0	0	0	1	0	0	0	0	0	0	1
14-Dec	4	1	2	0	0	0	1	0	0	0	0	0	0	0
14-Dec	4	1	3	0	0	0	1	0	0	0	0	0	0	1
14-Dec	4	1	4	0	0	0	1	0	0	0	1	0	0	1
14-Dec	4	1	5	0	0	0	1	0	0	0	0	0	0	1
14-Dec	4	1	6	0	0	0	1	0	0	0	1	0	0	1
14-Dec	4	1	7	0	0	0	1	0	0	0	0	0	0	6
14-Dec	4	1	8	0	0	0	1	0	0	0	0	0	0	1
14-Dec	4	1	9	0	0	0	1	0	0	0	0	0	0	1

14-Dec	4	1	10	0	0	0	1	0	0	0	0	0	0	1
14-Dec	4	1	11	0	0	0	1	0	0	0	0	0	0	2
14-Dec	4	1	12	0	0	0	1	0	0	0	0	0	0	1
14-Dec	4	2	13	0	0	0	1	0	0	0	1	0	0	6
14-Dec	4	2	14	0	0	0	1	0	0	0	0	0	0	2
14-Dec	4	2	15	0	0	0	1	0	0	0	0	0	0	1
14-Dec	4	2	16	0	0	0	1	0	0	0	0	0	0	2
14-Dec	4	2	17	0	0	0	1	0	0	0	0	0	0	2
14-Dec	4	2	18	0	0	0	1	0	0	0	1	0	0	2
14-Dec	4	2	19	0	0	0	1	0	0	0	0	0	0	2
14-Dec	4	2	20	0	0	0	1	0	0	0	0	0	0	4
14-Dec	4	2	21	0	0	0	1	0	0	0	0	0	0	1
14-Dec	4	2	22	0	0	0	1	0	0	0	1	0	0	2
14-Dec	4	2	23	0	0	0	1	0	0	0	0	0	0	1
14-Dec	4	2	24	0	0	0	2	0	0	0	0	0	0	2
15-Dec	4	3	25	0	0	0	1	0	0	0	0	0	0	6
15-Dec	4	3	26	0	0	0	1	0	0	0	0	0	0	1
15-Dec	4	3	27	0	0	0	1	0	0	0	0	0	0	3
15-Dec	4	3	28	0	0	0	1	0	0	0	0	0	0	2
15-Dec	4	3	29	0	0	0	1	0	0	0	0	0	0	4
15-Dec	4	3	30	0	0	0	1	0	0	1	0	0	0	4
15-Dec	4	3	31	0	0	0	1	0	0	0	0	0	0	4
15-Dec	4	3	32	0	0	0	1	0	0	0	0	0	0	2
15-Dec	4	3	33	0	0	0	1	0	0	0	1	1	0	2
15-Dec	4	3	34	0	0	1	1	0	0	0	1	0	0	3
15-Dec	4	3	35	0	0	0	1	0	0	0	0	0	0	2
15-Dec	4	3	36	0	0	1	1	0	0	0	0	0	0	2
15-Dec	4	4	37	0	0	0	1	0	0	0	0	0	0	1
15-Dec	4	4	38	0	0	0	1	0	0	0	1	0	0	2
15-Dec	4	4	39	0	0	0	1	0	0	0	0	0	0	4

15-Dec	4	4	40	0	0	0	1	0	0	0	0	1	0	4
15-Dec	4	4	41	0	0	0	1	0	0	0	1	0	0	1
15-Dec	4	4	42	0	0	0	1	0	0	0	0	0	0	4
15-Dec	4	4	43	0	0	0	1	0	0	0	1	0	0	0
15-Dec	4	4	44	0	0	0	1	0	0	0	0	0	0	1
15-Dec	4	4	45	0	0	0	1	0	0	0	1	0	0	1
15-Dec	4	4	46	0	0	0	1	0	0	0	1	0	0	0
15-Dec	4	4	47	0	0	0	1	0	0	0	0	0	0	3
15-Dec	4	4	48	0	0	0	2	0	0	0	1	0	0	1
4-Jan	7	1	1	0	0	1	1	0	0	0	1	0	1	4
4-Jan	7	1	2	0	0	1	1	0	0	0	0	0	1	4
4-Jan	7	1	3	0	0	0	1	0	0	0	1	0	1	4
4-Jan	7	1	4	0	0	0	1	0	0	0	0	0	1	5
4-Jan	7	1	5	0	0	1	2	0	0	0	1	0	2	5
4-Jan	7	1	6	0	0	1	1	0	0	0	1	0	1	4
4-Jan	7	1	7	0	0	1	1	0	0	0	0	0	1	4
4-Jan	7	1	8	0	0	1	1	0	0	0	0	0	1	4
4-Jan	7	1	9	0	0	0	1	0	0	0	0	0	1	4
4-Jan	7	1	10	0	0	1	1	0	0	0	1	0	1	4
4-Jan	7	1	11	0	0	1	1	0	0	0	0	0	1	4
4-Jan	7	1	12	0	0	1	1	0	0	0	0	1	1	4
4-Jan	7	2	13	0	0	1	1	0	1	0	1	0	2	4
4-Jan	7	2	14	0	0	0	1	0	0	0	0	0	1	4
4-Jan	7	2	15	0	0	1	1	0	0	0	0	0	1	4
4-Jan	7	2	16	0	0	1	1	0	0	0	0	0	1	6
4-Jan	7	2	17	0	0	0	1	0	0	0	1	0	1	4
4-Jan	7	2	18	0	0	1	1	0	0	0	1	0	2	5
4-Jan	7	2	19	0	0	2	1	0	0	0	1	0	1	4
4-Jan	7	2	20	0	0	2	1	0	0	0	1	0	1	6
4-Jan	7	2	21	0	0	1	1	0	0	0	0	0	1	5

4-Jan	7	2	22	0	0	0	1	0	0	0	0	0	2	4
4-Jan	7	2	23	0	0	0	1	0	0	0	0	0	1	4
4-Jan	7	2	24	0	0	1	1	0	0	0	0	0	1	4
4-Jan	7	3	25	0	0	1	1	0	0	0	0	0	2	5
4-Jan	7	3	26	0	0	1	1	0	0	0	0	0	1	4
4-Jan	7	3	27	0	0	1	1	0	0	0	0	0	2	6
4-Jan	7	3	28	0	0	1	1	0	0	0	0	0	2	6
4-Jan	7	3	29	0	0	1	1	0	0	0	0	0	2	5
4-Jan	7	3	30	0	0	1	1	0	0	1	1	2	2	7
4-Jan	7	3	31	0	0	1	1	0	0	0	0	0	1	6
4-Jan	7	3	32	0	0	0	1	0	0	0	0	1	2	6
4-Jan	7	3	33	0	0	2	1	0	0	0	1	0	2	5
4-Jan	7	3	34	0	0	1	1	0	0	0	1	1	2	7
4-Jan	7	3	35	0	0	2	1	0	1	0	0	0	2	7
4-Jan	7	3	36	0	0	1	1	0	0	0	0	1	2	6
4-Jan	7	4	37	0	0	2	1	0	0	0	0	0	1	5
4-Jan	7	4	38	0	0	1	1	0	0	0	1	0	1	5
4-Jan	7	4	39	0	0	1	1	0	0	0	0	0	1	6
4-Jan	7	4	40	0	0	0	1	0	0	0	0	0	1	4
4-Jan	7	4	41	0	0	1	1	0	0	0	1	0	1	5
4-Jan	7	4	42	0	0	1	1	0	0	0	0	0	1	4
4-Jan	7	4	43	0	0	1	1	0	0	0	0	1	1	4
4-Jan	7	4	44	0	0	1	1	0	0	0	0	0	1	4
4-Jan	7	4	45	0	0	1	1	0	0	0	0	0	1	5
4-Jan	7	4	46	0	0	1	1	0	0	0	0	1	1	6
4-Jan	7	4	47	0	0	0	1	0	0	0	1	0	1	6
4-Jan	7	4	48	0	0	0	1	0	0	0	0	0	1	4
9-Feb	12	1	1	0	0	0	1	0	0	0	0	0	0	0
9-Feb	12	1	2	0	0	1	1	0	0	0	0	0	0	0
9-Feb	12	1	3	0	0	0	1	0	0	0	0	1	0	0

9-Feb	12	1	4	0	0	0	1	0	0	0	0	0	0	0
9-Feb	12	1	5	0	0	0	2	0	0	0	1	0	0	0
9-Feb	12	1	6	0	0	1	1	0	0	0	1	0	0	0
9-Feb	12	1	7	0	0	0	1	0	0	0	1	0	0	0
9-Feb	12	1	8	0	0	0	1	0	0	0	0	0	0	0
9-Feb	12	1	9	0	0	1	1	0	0	0	0	0	0	0
9-Feb	12	1	10	0	0	0	1	0	0	0	0	0	0	0
9-Feb	12	1	11	0	0	0	2	0	0	0	1	0	0	0
9-Feb	12	1	12	0	0	0	1	0	0	0	0	0	0	0
9-Feb	12	2	13	0	0	0	1	0	0	0	1	0	0	0
9-Feb	12	2	14	0	0	0	1	0	0	0	0	0	0	0
9-Feb	12	2	15	0	0	0	1	0	0	0	0	0	0	0
9-Feb	12	2	16	0	0	0	1	0	0	0	1	0	0	0
9-Feb	12	2	17	0	0	0	1	0	0	0	0	0	0	0
9-Feb	12	2	18	0	0	0	1	0	0	0	1	0	0	0
9-Feb	12	2	19	0	0	0	1	0	0	0	1	0	0	0
9-Feb	12	2	20	0	0	1	1	0	0	0	0	0	0	0
9-Feb	12	2	21	0	0	0	1	0	0	0	1	0	0	0
9-Feb	12	2	22	0	0	0	1	0	0	0	0	0	0	0
9-Feb	12	2	23	0	0	0	1	0	0	0	0	0	0	0
9-Feb	12	2	24	0	0	1	1	0	0	0	0	0	0	0
9-Feb	12	3	25	0	0	0	1	0	0	0	0	0	0	0
9-Feb	12	3	26	0	0	0	2	0	0	0	0	0	0	0
9-Feb	12	3	27	0	0	0	1	0	0	0	0	0	0	0
9-Feb	12	3	28	0	0	0	1	0	0	0	0	0	0	0
9-Feb	12	3	29	0	0	1	1	0	0	0	1	0	0	0
~~9-Feb~~	~~12~~	~~3~~	~~30~~	~~0~~	~~0~~	~~0~~	~~1~~	~~0~~	~~0~~	~~0~~	~~0~~	~~0~~	~~0~~	~~0~~
9-Feb	12	3	31	0	0	0	1	0	0	0	0	0	0	0
9-Feb	12	3	32	0	0	0	1	0	0	0	0	0	0	0
9-Feb	12	3	33	0	0	0	1	0	0	0	1	0	0	0

9-Feb	12	3	34	0	0	0	1	0	0	0	1	0	0	0
9-Feb	12	3	35	0	0	1	1	0	0	0	0	0	0	0
9-Feb	12	3	36	0	0	0	1	0	0	0	0	0	0	0
9-Feb	12	4	37	0	0	0	1	0	0	0	1	0	0	0
9-Feb	12	4	38	0	0	0	1	0	0	0	1	0	0	0
9-Feb	12	4	39	0	0	0	1	0	0	0	0	0	0	0
9-Feb	12	4	40	0	0	0	2	0	0	0	0	0	0	0
9-Feb	12	4	41	0	0	0	1	0	0	0	1	0	0	0
9-Feb	12	4	42	0	0	0	1	0	0	0	0	0	0	0
9-Feb	12	4	43	0	0	0	1	0	0	0	1	0	0	0
9-Feb	12	4	44	0	0	0	1	0	0	0	0	0	0	0
9-Feb	12	4	45	0	0	0	1	0	0	0	0	0	0	0
9-Feb	12	4	46	0	0	0	1	0	0	0	1	0	0	0
9-Feb	12	4	47	0	0	0	1	0	0	0	1	0	0	0
9-Feb	12	4	48	0	0	0	2	0	0	0	0	0	0	0
29-Mar	19	1	1	0	0	1	1	0	0	0	1	0	0	0
29-Mar	19	1	2	0	0	2	1	0	0	0	1	0	0	0
29-Mar	19	1	3	0	0	1	1	0	0	0	0	1	0	0
29-Mar	19	1	4	0	0	1	2	0	0	0	0	0	0	0
29-Mar	19	1	5	0	0	1	2	0	0	0	1	1	0	0
29-Mar	19	1	6	0	0	0	1	0	0	0	1	0	0	0
29-Mar	19	1	7	0	0	1	1	0	0	0	0	0	0	0
29-Mar	19	1	8	0	0	2	1	0	0	0	0	0	0	0
29-Mar	19	1	9	0	0	0	1	0	0	0	0	0	0	0
29-Mar	19	1	10	0	0	2	1	0	0	0	1	0	0	0
29-Mar	19	1	11	0	0	1	1	0	1	0	0	0	0	0
29-Mar	19	1	12	0	0	0	1	0	0	0	0	0	0	0
29-Mar	19	2	13	0	0	0	1	0	1	0	1	0	0	0
29-Mar	19	2	14	0	0	0	1	0	0	0	0	0	1	0
29-Mar	19	2	15	0	0	1	1	0	0	0	1	0	0	0

29-Mar	19	2	16	0	0	1	1	0	0	0	0	0	0	0
29-Mar	19	2	17	0	0	1	1	0	0	0	0	0	0	0
29-Mar	19	2	18	0	0	0	1	0	0	0	0	0	0	0
29-Mar	19	2	19	0	0	1	1	0	0	0	0	0	0	0
29-Mar	19	2	20	0	0	0	1	0	0	0	1	0	0	0
29-Mar	19	2	21	0	0	0	2	0	0	0	0	0	0	0
29-Mar	19	2	22	0	0	1	1	0	1	1	0	0	0	0
29-Mar	19	2	23	0	0	0	1	0	0	0	0	0	0	0
29-Mar	19	2	24	0	0	1	2	0	0	0	0	0	0	0
29-Mar	19	3	25	0	0	0	1	0	0	0	0	0	1	4
29-Mar	19	3	26	0	0	0	1	0	0	0	0	0	2	5
29-Mar	19	3	27	0	0	0	1	0	0	0	0	0	1	4
29-Mar	19	3	28	0	0	0	1	0	0	0	0	0	2	4
29-Mar	19	3	29	0	0	1	2	0	0	0	1	0	2	5
29-Mar	19	3	31	0	0	0	1	0	0	0	0	0	1	3
29-Mar	19	3	32	0	0	0	1	0	1	0	0	0	1	2
29-Mar	19	3	33	0	0	0	1	0	0	0	1	0	1	4
29-Mar	19	3	34	0	0	1	1	0	0	0	1	0	0	4
29-Mar	19	3	35	0	0	0	1	0	0	0	0	1	0	4
29-Mar	19	3	36	0	0	0	1	0	0	0	0	0	2	2
29-Mar	19	4	37	0	0	0	1	0	0	0	1	0	2	4
29-Mar	19	4	38	0	0	0	1	0	0	0	1	0	2	3
29-Mar	19	4	39	0	0	0	2	0	0	0	0	0	1	3
29-Mar	19	4	40	0	0	0	1	0	1	0	1	0	1	4
29-Mar	19	4	41	0	0	0	1	0	1	0	1	0	2	5
29-Mar	19	4	42	0	0	2	1	0	0	0	0	0	1	2
29-Mar	19	4	43	0	0	0	1	0	0	0	1	0	1	3
29-Mar	19	4	44	0	0	2	1	0	0	0	0	0	1	3
29-Mar	19	4	45	0	0	0	1	0	0	0	1	0	1	4
29-Mar	19	4	46	0	0	0	1	0	1	0	0	0	2	4

29-Mar	19	4	47	0	0	0	1	0	0	0	0	0	2	4
29-Mar	19	4	48	0	0	1	2	0	1	0	0	0	1	2
